# Targeting neddylation and sumoylation in chemoresistant triple negative breast cancer

**DOI:** 10.1038/s41523-024-00644-4

**Published:** 2024-05-27

**Authors:** Reid T. Powell, Amanda L. Rinkenbaugh, Lei Guo, Shirong Cai, Jiansu Shao, Xinhui Zhou, Xiaomei Zhang, Sabrina Jeter-Jones, Chunxiao Fu, Yuan Qi, Faiza Baameur Hancock, Jason B. White, Clifford Stephan, Peter J. Davies, Stacy Moulder, W. Fraser Symmans, Jeffrey T. Chang, Helen Piwnica-Worms

**Affiliations:** 1 Center for Translational Cancer Research, Institute of Bioscience and Technology Texas A&M Health Science Center, Houston, TX USA; 2 Department of Experimental Radiation Oncology, The University of Texas MD Anderson Cancer Center, Houston, TX USA; 3 Department of Pathology, The University of Texas MD Anderson Cancer Center, Houston, TX USA; 4 Department of Bioinformatics and Computational Biology, The University of Texas MD Anderson Cancer Center, Houston, TX USA; 5 Department of Breast Medical Oncology, The University of Texas MD Anderson Cancer Center, Houston, TX USA; 6 Department of Integrative Biology and Pharmacology, The University of Texas Health Science Center at Houston, Houston, TX USA; 7 Present Address: Eli Lilly and Company, Indianapolis, IN USA

**Keywords:** Breast cancer, Targeted therapies

## Abstract

Triple negative breast cancer (TNBC) accounts for 15–20% of breast cancer cases in the United States. Systemic neoadjuvant chemotherapy (NACT), with or without immunotherapy, is the current standard of care for patients with early-stage TNBC. However, up to 70% of TNBC patients have significant residual disease once NACT is completed, which is associated with a high risk of developing recurrence within two to three years of surgical resection. To identify targetable vulnerabilities in chemoresistant TNBC, we generated longitudinal patient-derived xenograft (PDX) models from TNBC tumors before and after patients received NACT. We then compiled transcriptomes and drug response profiles for all models. Transcriptomic analysis identified the enrichment of aberrant protein homeostasis pathways in models from post-NACT tumors relative to pre-NACT tumors. This observation correlated with increased sensitivity in vitro to inhibitors targeting the proteasome, heat shock proteins, and neddylation pathways. Pevonedistat, a drug annotated as a NEDD8-activating enzyme (NAE) inhibitor, was prioritized for validation in vivo and demonstrated efficacy as a single agent in multiple PDX models of TNBC. Pharmacotranscriptomic analysis identified a pathway-level correlation between pevonedistat activity and post-translational modification (PTM) machinery, particularly involving neddylation and sumoylation targets. Elevated levels of both NEDD8 and SUMO1 were observed in models exhibiting a favorable response to pevonedistat compared to those with a less favorable response in vivo. Moreover, a correlation emerged between the expression of neddylation-regulated pathways and tumor response to pevonedistat, indicating that targeting these PTM pathways may prove effective in combating chemoresistant TNBC.

## Introduction

TNBC is a subtype of breast cancer that lacks expression of the estrogen and progesterone receptors and does not overproduce epidermal growth factor receptor 2 (HER2)^1^. The current standard of care for patients with early-stage TNBC consists of combinations of DNA-damaging agents (anthracyclines, phosphoramide mustards, platinum salts) and mitotic inhibitors (taxanes)^1–3^. Recently, immunotherapy has been approved for treating TNBC in the neoadjuvant setting, and PARP inhibitors are increasingly used for germline BRCA-mutant tumors, though still in the adjuvant setting^4–6^. While approximately one-third to one-half of TNBC patients receiving NACT achieve either a complete or partial response, the remainder of patients harbor significant residual cancer burden at the time of surgery, and this is associated with a high risk of recurrence or metastasis within two years of surgical resection^7^. Thus, identifying targetable vulnerabilities in chemoresistant TNBC remains an unmet clinical need.

High throughput drug screening identifies therapeutic vulnerabilities in cells derived from various tumor types. Large-scale high throughput screens (HTS), such as the Cancer Cell Line Encyclopedia (CCLE)^8^, Cancer Target Discovery and Development (CTD2), and the Genomics of Drug Sensitivity in Cancer (GDSC)^9^, have historically identified multiple drug targets and mechanisms associated with the development and progression of cancer. The coupling of large-scale HTS with genomic, transcriptomic, proteomic, and metabolomic information has refined the impact of screening studies and has been effectively leveraged to identify drug-specific biomarkers^10^, define mechanisms of action^11^, and develop predictive algorithms for personalized medicine applications^12^. A limitation in some of these HTS studies has been their reliance on established cell lines that do not fully recapitulate the extent of heterogeneity in patient-derived tumors. In response to this limitation, pre-clinical analysis of tumor-specific therapeutic vulnerabilities are increasingly using patient-derived tumor cells and model systems.

To identify therapeutic vulnerabilities, we developed a pipeline for transcriptomics and high throughput drug susceptibility profiling using cells from orthotopic PDX models. Previously, we applied this approach to cells isolated from sixteen treatment-naïve PDX models representing multiple TNBC subtypes and identified both pan-active and subtype-specific drugs^13^. This study interrogates cells derived from paired pre-, mid-, and post-NACT PDX models to identify targetable vulnerabilities in chemoresistant TNBC tumors. Our findings revealed that mid- and post-NACT tumors exhibited heightened sensitivity in vitro to drugs targeting protein homeostasis pathways. Pre-clinical studies further confirmed the effectiveness of pevonedistat as a single agent in treating TNBC.

## Results

### Generation of longitudinal TNBC PDX models

**Fig. 1:**
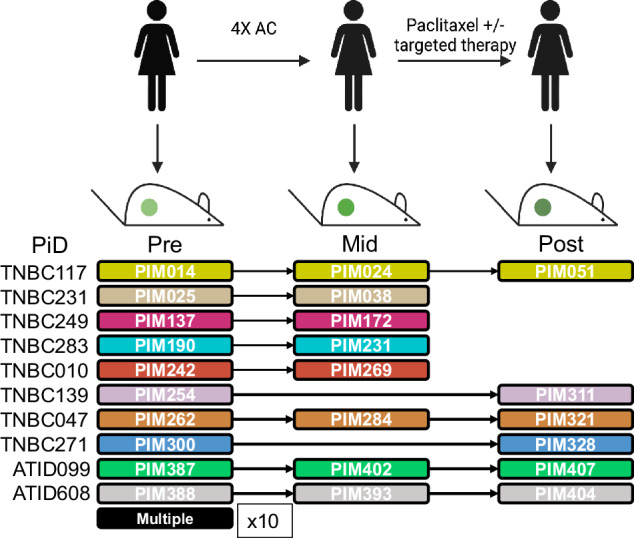
Study design. Schematic representation of the ARTEMIS clinical trial showing PDX models established at clinical presentation (Pre), after four cycles of Adriamycin and cyclophosphamide (AC, Mid), and after completion of NACT (Post). Patient IDs (PiD) and Patient in Mouse (PIM) IDs that were established at each time point are shown below. Color code denotes matched patient/PDX serial models.

As part of the prospective neoadjuvant ARTEMIS trial (NCT02276443)^13–15^, we obtained fine needle aspirates or core biopsies at critical clinical decision-making time points (Fig. 1) including clinical presentation before NACT (denoted as *pre*), from residual tumors after four cycles of Adriamycin (doxorubicin) and cyclophosphamide (AC; timepoint denoted as *mid*), and at surgical resection of residual tumors after paclitaxel ± targeted therapy (denoted as *post*). The cohort consists of 34 PDX profiles, eighteen more than we previously reported^13^, and now includes ten longitudinal sets (Fig. 1, Supplementary Fig. 1). Six pre-NACT models from the initial publication were expanded with longitudinal information, while four represent newly screened sets. Molecular subtyping analysis^16^ of this cohort revealed a representative sampling of TNBC subtypes with eight basal-like 1 (BL1), six basal-like 2 (BL2), five luminal androgen receptor (LAR), six mesenchymal (M), and nine unstable (UNS) when using the 4-class TNBCtype model^16^. Four of the longitudinal models altered molecular subtypes with treatment, two of which (TNBC283 & TNBC047) switched to the M subtype following NACT (Supplementary Fig. 1). An additional two sets (TNBC117 and TNBC010) had an increasingly significant association with the M subtype (Supplementary Fig. 2), but not to the extent of switching the overall classification.

### Curation of high throughput drug screening results

We performed unbiased high throughput drug viability screens on this panel of 34 PDX models using a library of 618 mechanistically annotated probes and oncology drugs (Supplementary Table 1). For these screens, freshly isolated tumors were depleted of mouse cells and transferred to 384-well plates, where they were cultured in serum-free Mammocult media. After 72 h of incubation with the drug libraries, viability was assessed using CellTiter Glo (CTG). We calculated the percent inhibition relative to DMSO control and fit the concentration response across the tested range (0.1, 1.0, and 10 μM) to derive area under the curve (AUC) values used during downstream analysis. Next, we applied a series of subjective filters to remove drugs that were either pan-active or pan-inactive by applying a threshold on the observed range of AUC values (RANGE ≥ 0.5), followed by removing potential growth-confounded drug responses (Growth index versus AUC r^*2*^ > 0.25) (Supplementary Fig. 3). In total, 145 drugs demonstrated a heterogenous, non-growth correlated response and were appropriate for downstream analysis.

### Differential drug susceptibility of matched PDX sets

**Fig. 2:**
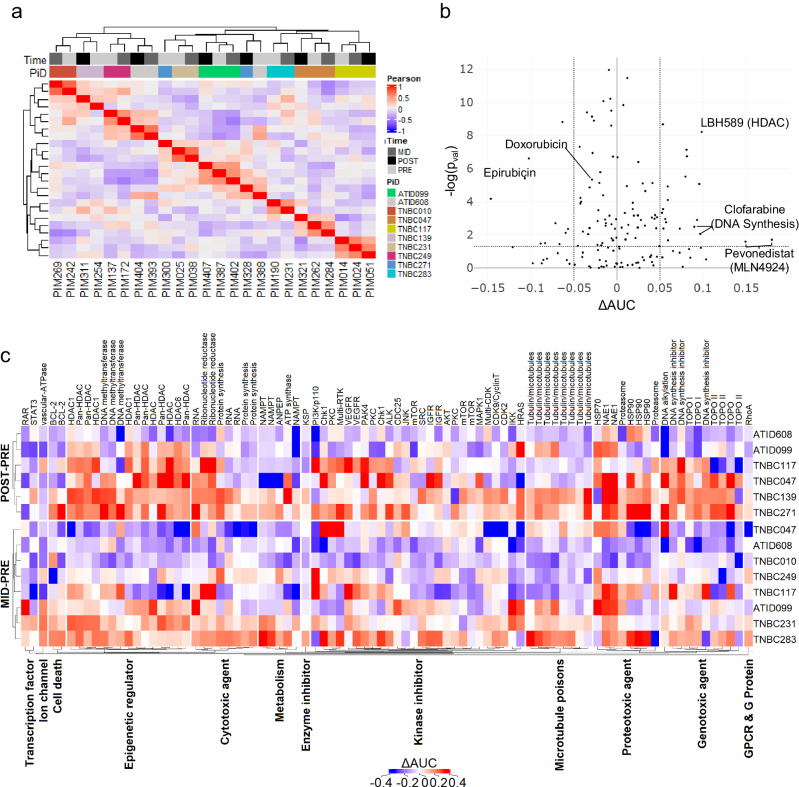
Longitudinal analysis of drug response profiles. **a** Heatmap showing the pairwise-Pearson correlation of cell lines using the z-normalized AUCs of the filtered drug profile. Top bar identifies the timepoint (Time) and Patient ID (PiD) using the color code denoted in the figure. **b** Volcano plot of the mean difference in the AUC (Time_x_-T_0_) by the log significance of the interaction determined from the linear model. The top 5 drugs are highlighted, in addition to components of the NACT regimen, which show an acquired resistance. Dotted lines show cut offs for ${-\log }_{10}\left(p-{value}\right)\,\ge 1.3\& \; \mathrm{abs}({mean}\left(\Delta {AUC}\right))\ge 0.05$) used to prioritize the top compounds from the screen. **c** Heat map showing the shifts in AUC values for each matched pair. Y-axis shows the Patient ID number grouped by Mid-to-Pre and Post-to-Pre comparisons. X-axis is grouped according to pre-established mechanistic class and annotated by target.

We then performed hierarchical clustering on the pharmacologic profiles of PDX-derived cancer cells and found that they segregated by the patient of origin (χ^2^ *p* value = 1.99 × 10^–7^, Fig. 2a), demonstrating that patient-level heterogeneity drove drug response profiles to a greater degree than therapy-induced changes. We observed two exceptions to patient-level co-clustering where the pre-treatment tumors, PIM300 and PIM388, separated from their corresponding mid-NACT and post-NACT treated tumors, PIM328 and PIM393/PIM404, respectively. To better define what drove these differences, we correlated the AUCs between matched pairs and saw a high overall correlation in drug susceptibility (AUC) with substantial variability in a small subset of drugs, as discussed below (Supplementary Fig. 4a, d). A more granular analysis of the correlation between technical replicates across assay plates (Supplementary Fig. 4b, c, e–g) showed a low correlation in a subset of wells from an isolated region of the assay plate for PIM328 (Supplementary Fig. 4c), when compared to its pre-treatment pair PIM300 (Supplementary Fig. 4b). Conversely, plate effects were not detected in the technical replicates of pre-treatment model PIM388 (Supplementary Fig. 4e). Thus, we conclude that the separation of PIM328 from its paired model is more likely driven by an experimental artifact, while the separation of PIM388 appears to be biologically driven.

Next, we looked for drugs with differential activity between the pre-NACT and mid/post-NACT time points using a linear response model that accounted for both the patient identifier and sample time point. An initial unpaired analysis to evaluate differential drug effects did not identify any significant changes. Accordingly, we moved to a paired analysis that assessed the consistency in the direction and magnitude of the ΔAUC between matched-pairs. From this analysis, we observed a subset of therapeutic targets that were repeatedly more effective in the mid/post-NACT model compared to their respective pre-NACT models (Fig. 2b, c). These analyses demonstrate the power of longitudinal follow-up when studying chemoresistance in the context of heterogeneous patient populations.

Drug classes that tended to become less active in mid-NACT or post-NACT versus pre-NACT PDX models included anthracyclines, taxanes, and multiple serine/threonine and receptor tyrosine kinase inhibitors (Supplementary Table 2). Importantly, mid-NACT models were established from tumors that showed poor responses to AC in patients, and post-NACT models were established from tumors that showed poor response to both AC (at mid-treatment) and the taxane paclitaxel ± additional therapies (at post-treatment). Conversely, tumor cells isolated from PDX models established from mid- and post-NACT tumors showed enhanced sensitivity relative to pre-NACT tumors to distinct drug classes, including epigenetic agents, DNA/RNA synthesis inhibitors, proteotoxic agents, and pro-apoptotic signaling drugs (Fig. 2b, c). Some of the genes and pathways targeted by these drug classes have been implicated in contributing to the development of chemoresistance. For example, BCL-2 inhibitors have been shown to re-sensitize TNBC cell lines to Adriamycin^17^, suggesting their potential use as a combination therapy with NACT in TNBC. Aberrant DNA methylation and histone acetylation have also been broadly implicated with resistance to multiple classes of chemotherapy^18,19^, which was consistent with our observation that DNA methyltransferase and histone deacetylase inhibitors showed increased activity in mid/post-NACT PDX models.

We next ranked drugs by their overall magnitude and statistical significance in conferring differential drug activity (mid vs pre or post vs pre). The top five drugs were pevonedistat and MLN4924, which are the same drug from two different sub-libraries and commercial sources and are annotated as an NAE1 inhibitor, followed by LBH589, a histone deacetylase (HDAC) inhibitor, and clofarabine, a DNA synthesis inhibitor also represented in two sub-libraries (Fig. 2b). In addition to pevonedistat and MLN4924, multiple additional proteotoxic agents (Proteasome: MG132, Carfilzomib; HSP: Elesclomol, NVP-AUY922, 17 DMAG, AT-13387) showed enhanced sensitivity in the mid/post-NACT models supporting a potential indication for the use of this drug class in treating chemoresistant TNBC. We prioritized pevonedistat for further investigation as it was the highest-ranking compound among the proteotoxic agents.

### Comparisons of longitudinal tumor transcriptomes

**Fig. 3:**
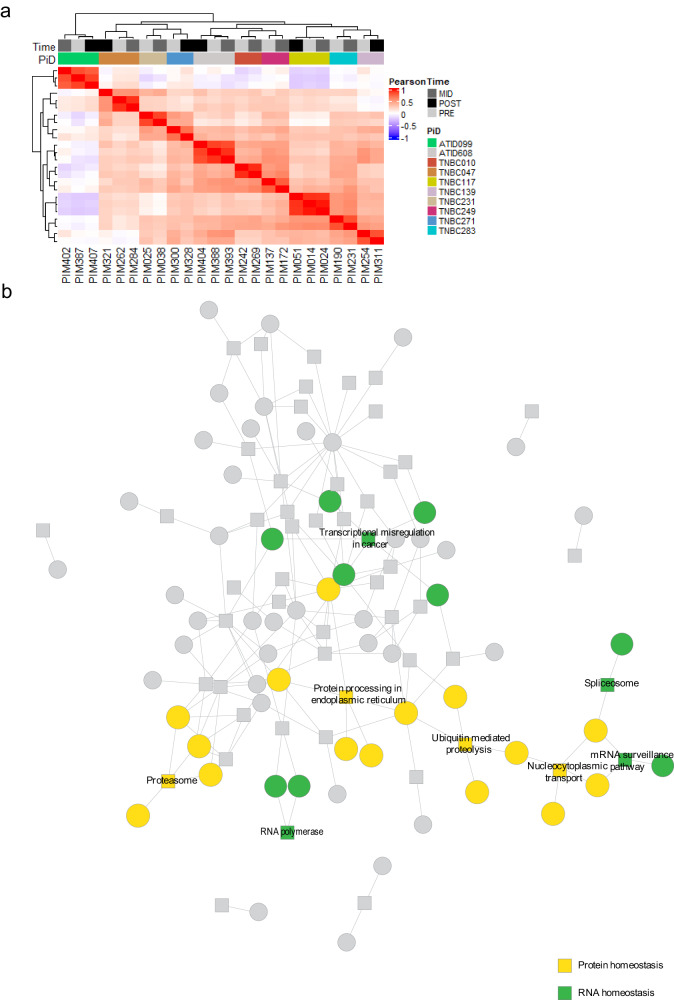
Longitudinal analysis of transcriptomic profiles. **a** Heatmap of the pairwise-Pearson correlation of cell lines using the z-normalized ComBat-adjusted TPM. Top bar denotes the timepoint and Patient ID using the color code denoted in the figure. **b** Heterogeneous network representation generated by performing gene-pathway enrichment analysis using pathfindR. Significantly altered genes are represented by circles, while pathway annotations are shown as squares, with connecting lines to member genes. Pathways and genes related to protein homeostasis are highlighted in yellow, while those related to RNA homeostasis are in green.

We performed a longitudinal analysis of gene expression profiles in parallel with the drug studies. From hierarchical clustering analysis, we found that pre- and post-treatment gene expression profiles from PDX tumors established from the same patient clustered together (Fig. 3a), recapitulating the clustering pattern seen in the drug response profiles (Fig. 2a). Next, we performed a differential gene expression analysis and identified 341 genes that were differentially expressed across time points (DEGs; Supplementary Table 3) when tumors from the same patients were paired, compared to only 6 DEGs when unpaired. Gene-pathway enrichment of the DEGs from the matched-pairs analysis identified an interconnected network that included multiple signaling pathways regulating protein homeostasis, including the KEGG^20^ spliceosome, proteasome, and ubiquitin-mediated proteolysis pathways (Fig. 3b). These data are further supported by an analysis of previously published RNA-seq data from a PDX model treated with AC therapy^21^, which showed significant enrichment of genes in the KEGG ubiquitin-mediated proteolysis and multiple sumoylation and neddylation pathways in residual tumor cells after AC when compared to vehicle (Supplementary Table 4). Thus, the transcriptomic data provide additional support for targeting protein homeostasis pathways in the post-NACT setting of chemoresistant TNBC.

### Unbiased analysis of pevonedistat activity

To discern the potential mechanisms of action for pevonedistat in this setting, we tested whether the association between protein modification and pevonedistat activity could be seen in previously published drug screening data sets (GDSC1 and CTD2), which we downloaded through the DepMap data portal (https://depmap.org/portal/). We first modeled the pevonedistat dose response using the AUC metric and trained a series of leave-one-out cross-validated (LOOCV) L1-penalized (lasso) linear regression models^22^. Each model incorporated genes from an individual C2 canonical pathway from the Molecular Signatures Database (MSigDB)^23^. The cross-validated predictive performance was then evaluated by minimizing the root mean squared error (RMSE) or maximizing the Pearson correlation to rank the predictive capability of individual pathways. When this analysis was applied to the CTD2 dataset^24^, “REACTOME_POST_TRANSLATIONAL_PROTEIN_MODIFICATION” was identified as the most predictive pathway (LOOCV Pearson R = 0.72, Supplementary Fig. 5a). Further feature importance, calculated as the absolute value of the coefficients of the tuned model, for genes within this pathway revealed enrichment of a narrow set of genes involved in neddylation and the deubiquitination machinery. This finding aligns well with established mechanisms of action for pevonedistat and serves to validate our approach. The “REACTOME_POST_TRANSLATIONAL_PROTEIN_MODIFICATION” pathway was also identified as the most predictive gene set (LOOCV Pearson R = 0.69) when the lasso regression analysis was applied to the GDSC1 dataset^9^ (Supplementary Fig. 5b). However, the top-ranked genes prioritized by this dataset also identified additional post-translational modifications including sumoylation. The fact that the top-ranked genes vary across data sets, despite belonging to the same pathway, underscores the ability of pathway analysis to provide a more robust interpretation of the active biological processes^25^.

Next, we performed the lasso regression analysis using only breast cancer data. Here, we identified “REACTOME_SUMOYLATION_OF_RNA_BINDING_PROTEINS”, “REACTOME_SUMOYLATION_OF_DNA_DAMAGE_RESPONSE_AND_REPAIR_PROTEINS”, “REACTOME_SUMOYLATION_OF_CHROMATIN_ORGANIZATION_PROTEINS”, and “KEGG_UBIQUITIN_MEDIATED_PROTEOLYSIS” using the CTD2 dataset (Supplementary Fig. 5c) and “REACTOME_SUMOYLATION_OF_UBIQUITINYLATION_PROTEINS”, “REACTOME_SUMOYLATION_OF_CHROMATIN_ORGANIZATION_PROTEINS”, and “REACTOME_SUMOYLATION_OF_DNA_REPLICATION_PROTEINS”, using the GDSC1 dataset (Supplementary Fig. 5d). It is important to note that the C2 canonical pathway gene set includes eighteen signatures associated with sumoylation, but only one for neddylation. As neddylation remains understudied compared to sumoylation, it is possible that we were not powered to capture additional facets of neddylation transcriptomic signatures.

When the LOOCV lasso regression analysis was applied to our dataset, we identified the closely related sub-pathway “REACTOME_SUMO_IS_CONJUGATED_TO_E_UBA2_SAE1” (LOOCV Pearson R = 0.44) and “BIOCARTA_SUMO_PATHWAY” (LOOCV Pearson R = 0.41) among the top 50 pathways that predicted pevonedistat activity (Supplementary Fig. 5e). Feature importance analysis of both pathways revealed a strong association between the expression levels of SAE1 and SUMO1 and pevonedistat activity in our dataset. A complete list of pathway information and feature importance analysis for all studies can be found on the Github page listed in the data availability section. Collectively, these data show an increasing relevance of the combined action of PTMs, including neddylation and sumoylation, in the activity relationship of pevonedistat response and TNBC. However, it should be noted that we were unable to find features that could robustly predict response across all datasets.

Pevonedistat studies conducted in TNBC cell lines in vitro revealed selective activity within a proteomic-defined TNBC subpopulation that significantly overlaps with the transcriptionally-defined BL1 TNBC^26^. We tested if the response to pevonedistat within our dataset correlated with distinct TNBC subtypes. Following Lehman et al.^16^ we evaluated the correlation between the continuous TNBCtype coefficients and drug response and found an association between pevonedistat activity and the BL1 and M TNBC subtypes (Supplementary Fig. 6).

### Pre-clinical study of pevonedistat in paired tumors

**Fig. 4:**
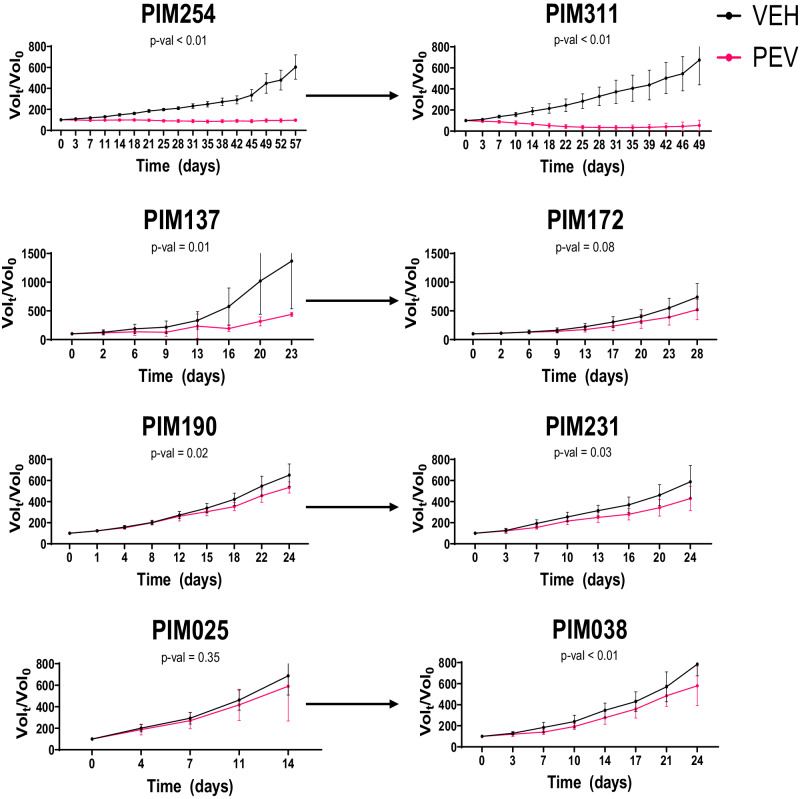
In vivo efficacy study of pevonedistat. Tumor volume growth curves for individual PDX models treated either with vehicle (black) or pevonedistat (red). Statistical significance denoted under the PDX_ID, was determined using a two-way ANOVA applied to the time series. Arrows connect paired models, with pre-NACT models on the left and corresponding mid/post-NACT models on the right. Data points and error bars show the mean and standard deviation respectively.

Both the drug screen and transcriptomic data suggested that protein homeostasis pathways represented a targetable vulnerability in TNBC following NACT. Based on these experimental observations, we prioritized pevonedistat for pre-clinical studies. We chose three longitudinal pairs (TNBC231: PIM025 and PIM038, TNBC283: PIM190 and PIM231, TNBC139: PIM254 and PIM311) that showed strong enhancement of pevonedistat response in mid- or post-NACT tumors relative to pre-NACT tumors in vitro and one longitudinal pair that showed robust response in both the pre-NACT and post-NACT settings (TNBC249: PIM137 and PIM172) (Supplementary Fig. 7). Tumor-bearing mice were treated daily with vehicle or 60 mg/kg pevonedistat^27^. This drug regimen was well-tolerated as there were no significant changes in activity or body weight between vehicle and pevonedistat-treated animals (Supplementary Fig. 8). Caliper measurements were taken twice weekly, and tumor volumes were calculated to evaluate the effect of pevonedistat on tumor growth. Three models had substantial responses to pevonedistat ranging from a two-thirds reduction of tumor volume (PIM311), no increase in tumor volume (PIM254), or a slowed increase in tumor volume (PIM137) relative to controls (Fig. 4). The remaining models exhibited less robust responses to pevonedistat, but in each case, slower increases in tumor volume on treatment were observed compared with the respective vehicle-treated controls. We continued pevonedistat treatment for up to three months in both PIM254 and PIM311 to determine the durability of the response. Resistance to pevonedistat was not observed in PIM254 tumors, but a subset of PIM311 tumors developed resistance within two months of treatment (Supplementary Fig. 9).

**Fig. 5:**
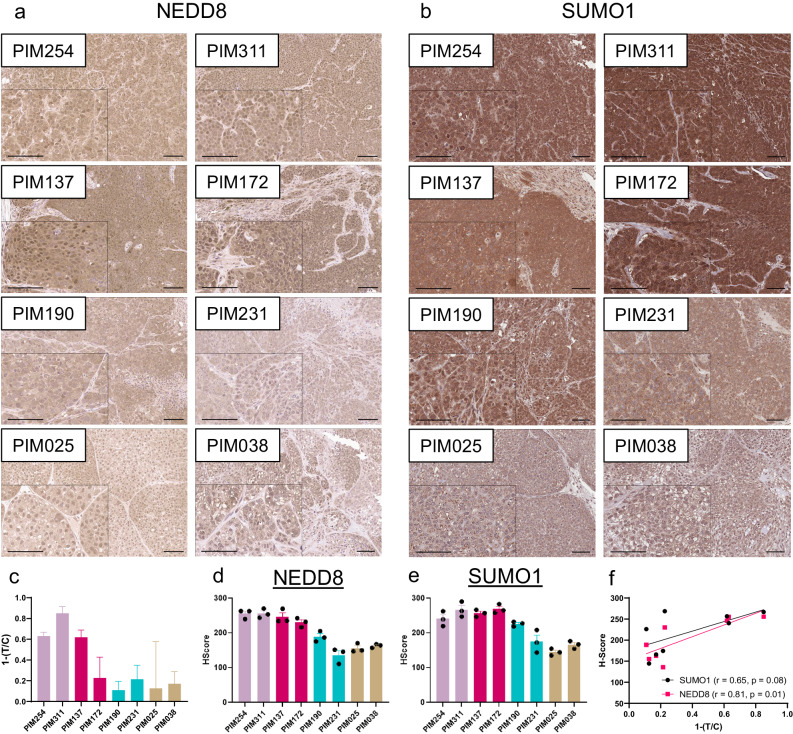
NEDD8 and SUMO1 tissue labeling. Representative images of NEDD8 (**a**) and SUMO1 (**b**) IHC in PDX tumor sections. Images shown are ×10 magnification, insets are ×20 magnification, and scale bars are 100 µm. **c** Bar chart showing PDX response to pevonedistat via normalized tumor to control ratios (T/C). Bars represent mean ± standard deviation. H-scores for NEDD8 (**d**) and SUMO1 (**e**) intensity, combining percent of tumor cells staining and intensity of staining. Images were quantified from three tumors per PDX model. Bars represent the mean ± SEM. **f** Dot plot showing the correlation of pevonedistat response (1-T/C) of SUMO1 H-Score (black) or NEDD8 H-score (Red). r = Spearman correlation coefficient, p = *p* value calculated from a two-tailed test.

Given the identification of both neddylation and sumoylation pathways in our transcriptomic analyses, we conducted immunohistochemistry to assess levels of NEDD8 and SUMO1 in PDX tumor samples (Fig. 5a, b). NEDD8 and SUMO1 levels were the highest in those tumors that responded to pevonedistat in vivo. Quantification of staining intensity via H-score similarly showed that the three models with the strongest response to pevonedistat, PIM254, PIM311, and PIM137, also had the three highest H-scores (Fig. 5c–e) and both NEDD8 and SUMO1 intensity was strongly correlated with response (Fig. 5f). While we could appreciate heterogeneity in the nuclear/cytoplasmic staining patterns within individual tumors, the general high-to-low trends of NEDD8 and SUMO1 abundance were maintained whether we quantified nuclear, cytoplasmic, or cellular staining intensity (Supplementary Fig. 10). An exception to this trend was PIM172, which showed comparable levels of NEDD8 and SUMO1 to those tumor models that responded robustly to pevonedistat in vivo.

### In vivo pharmacotranscriptomic analysis

To understand the relationship between gene expression and response in vivo, we correlated gene expression to the tumor to control ratio (T/C), described in methods, followed by gene set enrichment analysis on well-correlated genes. These analyses identified multiple genes belonging to the KEGG ubiquitin-mediated proteolysis pathway associated with pevonedistat response. Importantly, this gene set captures multiple components of post-translational modifications, including sumoylation and neddylation machinery (Supplementary Fig. 11), which is consistent with what was identified when analyzing the transcriptomes of the longitudinal PDX models (Fig. 3b), providing further evidence of the connection between this pathway and pevonedistat response in chemoresistant TNBC. Taken together, these findings suggest that the efficacy of pevonedistat may be attributed to inhibition of both the neddylation (NAE1) and sumoylation (SAE1)^28^ activating enzymes and/or through disruption of the crosstalk that occurs between neddylation and sumoylation pathways during a stress response^29^.

### Temporal analysis of in vivo pevonedistat response

**Fig. 6:**
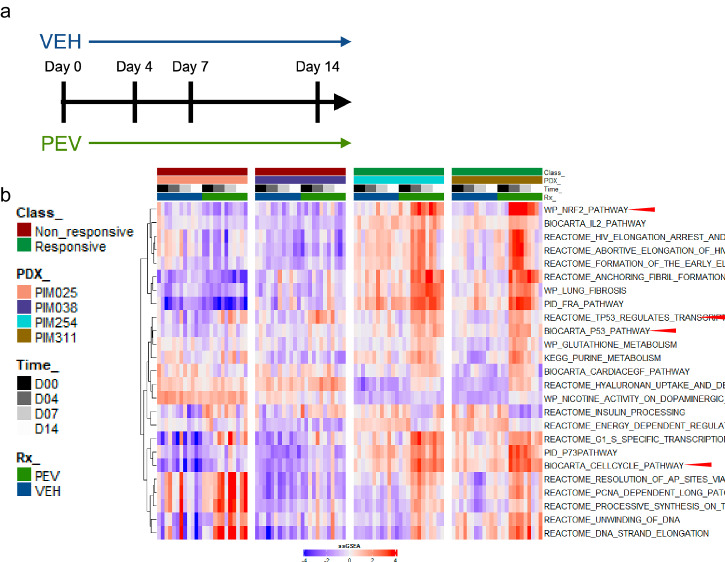
Perturbational response to pevonedistat in vivo. **a** Schematic of the FNA time series design. **b** Heatmap of the top ssGSEA pathways that showed a significant (*p* < 0.001) difference in the time series of the responsive class but not (*p* > 0.05) in the vehicle-treated controls. Top bar indicates class of pevonedistat response (responsive, non-responsive), PDX_ID, timepoint, and treatment.

To determine if downstream effectors of neddylation or sumoylation correlated with pevonedistat activity in vivo, we repeated our pre-clinical pevonedistat studies and collected fine needle aspirates (FNAs) from individual tumors as a function of time throughout treatment for one responsive pair of PDX models (PIM254 and PIM311) and one resistant pair (PIM025 and PIM038) (Fig. 6a). RNA sequencing was performed and subjected to single sample gene set enrichment analysis (ssGSEA) using the C2 canonical pathways from MSigDB. We then used a recursive feature elimination support vector machine (SVM) analysis^30^ to identify pathways where activity varied across the time series, and we identified multiple pathways known to be regulated by neddylation and sumoylation. These included upregulation of the NRF2 pathway, multiple cell cycle pathways, and pathways describing *TP53* regulation (Fig. 6b). Importantly, both NRF2 and Cdt1 stabilization are well-established responses to pevonedistat treatment^31,32^. Pevonedistat responsive pairs (PIM254 and PIM311) but not resistant pairs (PIM025 and PIM038) exhibited alterations in these pathways as a function of time following pevonedistat treatment.

## Discussion

In this study, we leveraged a unique collection of longitudinal PDX models to identify targetable vulnerabilities that emerge after neoadjuvant AC treatment for potential use in the treatment of chemoresistant TNBC. It should be noted that mice engrafted with patient tumors were never treated with AC, thus transcriptional changes and drug sensitivities identified in mid- and post-NACT tumors emerged during patient treatment and remained durable upon tumor engraftment and PDX establishment. From the combined longitudinal analyses of these models, we identified multiple tractable therapeutic targets for NACT-resistant TNBC. These included targeted drug classes that have been previously implicated in contributing to the development of chemoresistance. For example, BCL-2 inhibitors showed elevated susceptibility in post/mid-NACT models when compared to their pre-NACT counterparts in our data set. Consistently, BCL-2 inhibitors have also been shown to re-sensitize TNBC cell lines to Adriamycin^17^, suggesting their potential use as a combination therapy with NACT in TNBC. Similarly, aberrant DNA methylation and histone acetylation have been broadly implicated across multiple cancers with resistance to chemotherapy^18,19^. This is recapitulated in our dataset with the observation that DNA methyltransferase and histone deacetylase inhibitors showed increased activity in mid/post-NACT PDX models. We also made more novel observations with the identification of specific pathways that converged on protein homeostasis as consistently dysregulated after NACT. Here, we demonstrated the efficacy of pevonedistat as a single agent in a subset of PDX models, setting the stage for further validation of neddylation inhibition, along with exploration of sumoylation inhibition, in chemoresistant TNBC. Interestingly, others have found the NEDD8 pathway to be enriched in basal A breast cancer cell lines, and these cell lines were sensitive to NEDD8 depletion and inhibition in vitro^33^. A multi-omic analysis of TNBC patient tumor samples identified a vulnerability to pevonedistat in a proteomic-defined subpopulation that shares many attributes with BL1 TNBCtype tumors, which heavily overlaps with the basal A group, indicating a potential sensitivity in this particular subset of TNBC^26,34^.

Pevonedistat is a first-in-class drug that inhibits an enzymatic cascade that appends NEDD8 to substrate proteins. Under normal physiological circumstances, neddylation affects the stability, activity, and localization of a wide array of substrates to maintain cellular homeostasis. The most prominent substrates regulated by neddylation are the family of Cullin-RING ligases, components of the ubiquitin-proteasome system that undergo a conformational shift resulting in increased activity following neddylation. More recently, neddylation has been shown to target a wider array of proteins that are implicated in a broader range of cellular processes including mitochondrial fission/fusion cycles, metabolic reprogramming, ribosomal biogenesis, alternative splicing, and regulation of the tumor microenvironment^29,33,35–39^. In our studies, we tested pevonedistat as a single agent and found that it significantly reduced tumor volumes relative to vehicle treatment in three of the eight PDX models tested. As pevonedistat was identified through an emphasis on chemoresistant samples in our analysis, clinical utilization of pevonedistat would likely start in the setting of chemoresistant disease. As we understand more about the responsive patient population, there may be an avenue to explore pevonedistat as a combination therapy in the neoadjuvant setting. In particular, chemotherapy has been shown to induce proteotoxic stress in cancer cells^40,41^, while aberrant neddylation patterns along with hybrid neddylation-sumoylation modifications also arise during conditions of proteotoxic stress^29^. This interplay between neddylation and sumoylation could contribute to the identification of signatures associated with both pathways throughout our analyses. For any clinical development, it will be important to identify biomarkers predictive of response, which could start with further exploration of NEDD8 and SUMO1 protein levels in tumor tissues based on the results in our PDX models, and to explore mechanisms of pevonedistat resistance. *PTEN* loss has been implicated as a driver of pevonedistat resistance in breast cancer^42^.

By necessity, our PDX models were studied in immunocompromised mice. However, there is a body of literature that implicates neddylation in the function of various immune cell lineages. Studies have shown impaired proliferation, survival, and activation of T cells when neddylation is inhibited^43–46^. In other settings, neddylation abrogates the cytokine production and tumor infiltration of macrophages and myeloid-derived suppressor cells^39,47–49^. Given the complex nature of these pleiotropic interactions, further studies of pevonedistat in immune-competent models of TNBC are warranted to determine if potential microenvironmental effects could improve or hinder overall tumor responses. Taken together, our results show promise with pevonedistat in chemoresistant TNBC PDX models, providing the basis for future investigations to optimize therapeutic efficacy, better stratify responsive and resistant tumors, and explore potential synergistic combinations.

## Methods

### Collection of patient-derived materials

All research conducted in human patients followed national guidelines including the Common Rule (http://www.hhs.gov/ohrp/humansubjects/commonrule/), declaration of Helsinki (https://www.wma.net/policies-post/wma-declaration-of-helsinki-ethical-principles-for-medical-research-involving-human-subjects/) and the Health Insurance Portability and Accountability Act (HIPAA) privacy and security rules^50^. All patients from whom samples were collected for the generation of PDX models gave informed consent and were enrolled in the ARTEMIS trial (NCT02276443), an MD Anderson IRB-approved protocol (2014-0185).

### Animals

All experimental procedures were approved by the Institutional Animal Care and Use Committee (IACUC) at MD Anderson Cancer Center under IACUC protocol 00000978. Female NOD/SCID mice (NOD.CB17-Prkdcscid/NcrCrl) were obtained from Charles River, National Cancer Institute Colony. Endpoints for animal experiments were selected in accordance with IACUC-approved criteria, generally when tumors were 1.0–1.5 cm in diameter. Animals were humanely euthanized according to NIH and AAALAC guidelines, via carbon dioxide exposure followed by cervical dislocation.

### PDX cell preparation for drug screen

Cell preparation and quality control was performed as previously described^13^. In brief, the fourth mammary fat pads of 4-to-8-week-old female NOD/SCID mice were implanted with 500,000 PDX tumor cells, while the mice were anesthetized via isoflurane. Tumor cells were suspended in 20 µL of a 50:50 mixture of DMEM/F12 (HyClone, Cat. No. SH30023.01) media and Matrigel, (Corning, Cat. No. 354234) and then maintained on ice until engraftment. Mice received analgesics in the form of a subcutaneous 50 µL injection of 0.5 mg/mL extended-release buprenorphine (ZooPharm). Tumors were monitored weekly. When tumors reached about 1000 mm^3^, they were harvested and dissociated into single cells and organoids by mechanical mincing, followed by digestion with 3 mg/mL collagenase (Roche, Cat. No. 10103586001) and 0.6 mg/mL hyaluronidase (Sigma-Aldrich, Cat. No. H3506) supplemented with 2% bovine serum albumin (Sigma-Aldrich, Cat. No. A9418) in DMEM/F12 containing antibiotics (penicillin (100 U/mL), streptomycin (100 µg/mL), and amphotericin B (0.25 µg/mL)). Tumor digests were incubated on a rotating platform for 4 h at 37°C. Digested PDX tumor cells were re-suspended in red blood cell lysis buffer (Sigma, Cat. No. R7757), then treated with 0.25% Trypsin-EDTA (Corning, Cat. No. MT25053CI), followed by 5 U/mL Dispase (Stemcell Technologies, Cat. No. 07913) and 1 mg/mL DNase I solution (Stemcell Technologies, Cat. No. 07900). Finally, cells went through magnetic-activated cell sorting using the mouse cell depletion kit (Miltenyi Biotec, Cat. No. 130-104-694) to remove mouse cells. On average, 40 million PDX-derived tumor cells were isolated and subjected to the drug screening process per PDX model.

### Screening assays

Before plating, cell number and viability were determined by mixing 10 µL of culture media containing tumor cells with 10 µL trypan blue solution in a disposable counting slide, which was then read using a TC10 automated cell counter (Bio-Rad). Next, 2,000 viable cells/well were transferred into barcoded 384-well clear plates (Greiner, Cat No. 781091) using a MultiDrop Combi Reagent dispenser (Thermo). All drug libraries were diluted in DMSO and arrayed on Echo certified low dead volume plates (LDV, Labcyte). Drugs were subsequently transferred from the LDV source plate into assay plates using an Echo liquid handling machine (Labcyte). Wells were treated such that the final concentration of DMSO in media did not exceed 1% (v/v). Each assay plate had eight vehicle (DMSO) treated negative control wells, eight cytotoxic positive controls (10 μM Anisomycin), and an on-plate 8-point (10 μM to 4.6 nM in technical duplicate) dose response curve of the positive control. Screening assays were done in a single batch per PDX model with at least two off-plate technical replicates for library compounds, which were determined based on the availability and cellular viability of the starting materials provided.

### Screening rigor and reproducibility analysis

HTS are done in accordance with the NCATS assay guidance manual^51^ and as previously described^13^. In brief, we monitored the consistency and robustness of the 72-h CTG read-out by first evaluating the robust Z’ metric defined as:

1 $${Z}^{{\prime} }=1-\frac{3\left({{MAD}}_{{pos}}+{{MAD}}_{{neg}}\right)}{\left|{\widetilde{X}}_{{Pos}}{-\widetilde{X}}_{{Neg}}\right|}$$

where ${{MAD}}_{{pos}},{{MAD}}_{{neg}}$ are the median absolute distance of the positive (10 µM Anisomycin, *N* = 8) and negative (DMSO, *N* = 8) controls and ${\widetilde{X}}_{{Pos}}{,\widetilde{X}}_{{Neg}}$ are the median of the positive and negative controls. A Z’ > 0.5 is considered acceptable for continuous read-out assays. On-plate 8-point concentration response curves of Anisomycin are used to monitor reproducibility using the MSR statistic defined as:

2 $${MSR}={10}^{2\root2\of{2}{\sigma }_{{IC}50}}$$

Where ${\sigma }_{{IC}50}$ is the standard deviation in the IC50 values across assay plates. An MSR < 3 is generally considered to have sufficient reproducibility to perform quantitative activity-based analysis. All PDX models tested here had a median robust Z’ greater than 0.5 (Supplementary Fig. 12A, C). However, it was noted that PIM393 had markedly lower performance, which was determined to be the result of a transfer failure of the control compound. Similarly, we observed strong reproducibility with low MSR values, with only three MSR values greater than three (Supplementary Fig. 12B, C). Collectively, these data show high levels of robustness and technical reproducibility for screening assays.

### Administration of pevonedistat in vivo

PDX cells were implanted following the same protocol as for the drug screen above. Mice were randomly assigned to treatment or control arms and treatment was initiated when tumors reached ~100–150 mm^3^. Pevonedistat was formulated in 10% DMSO and 18% beta-cyclodextrin and dosed at 60 mg/kg by intraperitoneal injection daily. Vehicle control was dosed on the same schedule. Beta-cyclodextrin was made by adding 10 g (2-hydroxypropyl)-beta-cyclodextrin (Sigma, Cat No. C0926) to 50 mL sterile saline. (VWR, Cat No. 101320-574). Body weight and tumor size were measured 1–2 times per week. To make robust comparisons across heterogeneous PDX models, we normalized the tumor size for each timepoint to the starting tumor size for each individual mouse. Next, we fit the growth data using a generalized additive model (GAM) across the time series, to provide an outlier robust response metric. Finally, we numerically integrated the GAM function and reported the response as a time-integrated tumor to control ratio (T/C)^52,53^. Importantly, this approach leverages data from the entire time series, accounts for heterogenous growth effects, and has previously been shown to be more sensitive than conventional endpoint volumetric readouts^52,53^.

### Immunohistochemistry

Tumors were collected and incubated in 10% formalin at 4 °C for approximately 48–72 h for fixation, then embedded in paraffin. Formalin-fixed, paraffin-embedded (FFPE) tissues were sectioned by the MD Anderson Research Histology Core or the Center for Radiation Oncology Research Histology Core. FFPE slides were baked at 65 °C for one hour, then dewaxed and rehydrated by graded washes in xylene-to-ethanol. Heat-mediated epitope retrieval was performed by incubating slides in Reveal Decloaker (Biocare Medical, Cat. No. RV1000M), heated to 97 °C for 15 min using an EZ-Retriever microwave (BioGenex). Blocking of the slides used Dual Endogenous Enzyme Block (Dako, Cat. No. S200389-2) for 10 min, Protein Block (Dako, Cat. No. X0909) overnight at 4°C, and normal serum (Vector ImmPRESS, Cat. No. mp-7401) for 20 min. Slides were incubated with NEDD8 primary antibody (Cell Signaling, Cat. No. 2754, 1:250) or SUMO1 primary antibody (Cell Signaling, Cat. No. 4930, 1:50) for 1 h at RT, washed in PBS, then incubated with rabbit secondary antibody (Vector ImmPRESS, Cat. No. mp-7401). To develop the immunostain, slides were incubated with horse radish peroxidase substrate (HRP, VectorImmPACT, Cat. No. sk-4105), then counterstained with hematoxylin QS (Vector, Cat. No. H-3404), dehydrated through ethanol-to-xylene washes, and mounted using permanent mounting medium (VectraMount, Cat. No. H-5000).

### Immunohistochemistry analysis

Images were quantified using a custom workflow that leveraged a combination of ilastik^54^, Cell Pose^55^, and Pipeline Pilot (2023 Server Edition, Biovia). Here, ilastik was initially used to train a supervised stroma vs. tumor cell pixel classifier using expert-annotated images. The resulting model was then deployed to generate a probabilistic image with the same dimensions as the input and used to computationally deplete stromal cells from the analysis. Next, the RGB color input image was temporarily desaturated, inverted, and nuclear regions identified using the Cell Pose “nuclei” model with default settings. Nuclear regions were then expanded by a 10-pixel fixed radius to define rough cell boundaries. Xor logic was then used to generate cytoplasmic regions from the nuclear and cell mask regions. A pre-trained color space deconvolution algorithm from the advanced imaging collection of Pipeline Pilot was then used to separate purple/brown signal into pseudo-fluorescent images. Signal densitometry analysis was then performed for each regional boundary. Pipeline Pilot was used to integrate and automate the inputs/outputs between ilastik and Cell Pose and to calculate the final output metrics. Following quantification of staining intensity, cells were assigned to one of four bins (0–3+ ) based on the range of intensity values. H-scores were calculated using the formula 1*(%1cells) + 2*(%2cells) + 3*(%3cells), where the maximal score is 300.

### Collection of FNAs from PDX tumors

PDX cells were implanted, then tumors were assigned to treatment arms, dosed, and measured as above. At the indicated timepoints, FNAs were collected from the PDX tumors. Mice were anesthetized using isoflurane. A 23-gauge needle was inserted into the tumor through the skin and rapidly moved back and forth through the tumor mass at least 40 times. The material was then ejected from the needle into RNAlater (Invitrogen, Cat. No. AM7022) and stored for future RNA isolation. To sample various regions of each tumor, three separate passes were attempted for each tumor at each timepoint, and then pooled for all analyses.

### RNA isolation and sequencing

The collected FNA sample was placed into a 1.5 ml vial of RNAlater (Invitrogen, Cat. No. AM7022) at room temperature for up to one hour, then stored frozen at -80°C until use. The RNA was extracted using PicoPure RNA isolation kit (ThermoFisher Scientific, Cat. No. KIT0214) and RNA concentration was quantified by Nanodrop (Nanodrop Technologies). Whole transcriptome RNA-seq libraries were prepared using RNA HyperPrep kit with RiboErase (HMR) (Kapa Biosystems), following the manufacturer’s instructions. 100 bp paired-end sequencing was performed on NovaSeq 6000 using S4 Reagent Kit with 48 libraries pooled per lane.

### Generation of gene expression profiles

We processed the next-generation sequencing data using the BETSY system^56^. We called variants and estimated gene expression values as previously described^57^, except that we now identified contaminating mouse host reads for subtraction using Xenome^58^. The overall quality of the gene expression data was assessed using standard metrics^59^ (e.g., total read count, percent mapped reads; Supplementary Table 5). The gene expression data was sequenced in six separate batches and preprocessed separately using the same pipeline. We applied ComBat and PCA to normalize and monitor the presence of technical artifacts (Supplementary Fig. 13). Based on this approach, we found that three batches of the data had distinct gene expression profiles from the other three, and therefore normalized the three outlier batches against the other ones as background.

### Sample identity verification

We validated the identities of the RNA-seq profiles of the PDX samples using the mutations seen in the RNA-seq reads. After mapping reads according to the procedure above, we called mutations using the FreeBayes algorithm^60^ and compared them using NGSCheckMate^61^. We verified that the best matches (by correlation) of each tumor sample was against other PDX, patient tumor, or patient germline samples from the same patient (Supplementary Fig. 14).

### Statistics and software

DEG analysis was performed using the *limma*^62^ and *edgeR*^63^ and pathway enrichment analysis was performed using *pathfindR*^64^ R packages. Full details of the R environment and package versions are summarized at the end of Rmarkdown notebooks maintained on Github, see data availability section. High throughput drug screening data was analyzed using an automated screening tracking workflow developed in Pipeline Pilot (2023 Server, BIOVIA) and R statistics 4.1.2. The log concentration was fit to the normalized response using a cascade model which leverages the iteratively reweighted least squared method to fit the response surface to a four-parameter logistic or linear model. AUC and IC_50_ values were generated from the fitted dose response curve.

### Reporting summary

Further information on research design is available in the Nature Research Reporting Summary linked to this article.

### Supplementary information

Supplemental Figures

Reporting Summary

Supplemental Table 1) Chemical infromation for high throughput screens

Supplemental Table 2) Paired analysis of drug response

Supplemental Table 3) Paired DGE analysis

Supplemental Table 4) Retro-analysis of public A/C response data

Supplemental Table 5) RNAseq quality control metrics

## Data Availability

RNA sequencing data is available through the gene expression omnibus (https://www.ncbi.nlm.nih.gov/geo/) under the accession GSE264252. Curated data frames and additional metadata used to generate figures can be found at https://github.com/ReidTPowell/TNBC-PGx.

## Code availability

Open-source code, and interactive analytical outputs (e.g., networks) are available without restriction at https://github.com/ReidTPowell/TNBC-PGx. Code was developed using R 4.3.1 using RStudio. Specific package requirements and version can be found in the R markdown notebook on the listed page. Analytical workflows generated in commercial/proprietary environments (BioVia Pipeline Pilot) are available upon request.
