# Supplementary material for: Targeting neddylation and sumoylation in chemoresistant triple negative breast cancer
Source: NPJ Breast Cancer. 2024 May 27;10:37. doi: 10.1038/s41523-024-00644-4 (PMC11130334; doi:10.1038/s41523-024-00644-4)
Supplement: Supplementary file 1 — Supplemental Figures [file 41523_2024_644_MOESM1_ESM.pdf]

Supplemental Figure 1

Index	PDX ID	Time	PiD	*SciRep 2020*	Trial Drug	RCB Status	TNBCtype4
1	PIM010	PRE	TNBC044	X		RCB-II	M
2	PIM014	PRE	TNBC117	X		RCB-III	BL1
3	PIM024	MID	TNBC117			RCB-III	BL1
4	PIM051	POST	TNBC117		AA	RCB-III	BL1
5	PIM025	PRE	TNBC231	X		RCB-II	UNS
6	PIM038	MID	TNBC231			RCB-II	UNS
7	PIM040	PRE	TNBC081	X		RCB-II	LAR
8	PIM046	PRE	TNBC023	X		RCB-II	BL2
9	PIM053	PRE	TNBC069	X		RCB-I	LAR
10	PIM056	PRE	TNBC063	X		RCB-III	BL2
11	PIM068	PRE	TNBC016	X		pCR	M
12	PIM077	PRE	TNBC253	X		RCB-II	BL2
13	PIM091	PRE	TNBC052	X		RCB-III	LAR
14	PIM137	PRE	TNBC249	X		pCR	BL2
15	PIM172	MID	TNBC249			pCR	UNS
16	PIM163	PRE	TNBC192	X		RCB-III	BL1
17	PIM164	PRE	TNBC155	X		RCB-I	LAR
18	PIM190	PRE	TNBC283			RCB-II	BL1
19	PIM231	MID	TNBC283			RCB-II	M
20	PIM242	PRE	TNBC010	X		RCB-II	UNS
21	PIM269	MID	TNBC010			RCB-II	UNS
22	PIM254	PRE	TNBC139	X		RCB-II	UNS
23	PIM311	POST	TNBC139		P	RCB-II	BL2
24	PIM262	PRE	TNBC047	X		RCB-II	BL2
25	PIM284	MID	TNBC047			RCB-II	LAR
26	PIM321	POST	TNBC047		AA	RCB-II	M
27	PIM300	PRE	TNBC271			RCB-II	M
28	PIM328	POST	TNBC271		AA	RCB-II	M
29	PIM387	PRE	ATID099			RCB-III	UNS
30	PIM402	MID	ATID099			RCB-III	UNS
31	PIM407	POST	ATID099		PE	RCB-III	UNS
32	PIM388	PRE	ATID608			RCB-II	BL1
33	PIM393	MID	ATID608			RCB-II	BL1
34	PIM404	POST	ATID608		PCT	RCB-II	BL1

Supplemental Figure 1. Table of metadata parameters of the PDX models. PDX_ID – PDX model name. Time – timepoint in patient treatment from which the PDX model was derived. PiD – patient identifier. SciRep 2020 – X denotes if the sample was included in the initial Scientific Reports[13] manuscript. Trial Drug – for post-NACT samples, patients had received a range of treatments, abbreviated as: AA – atezolizumab and abraxane, P – paclitaxel, PE – paclitaxel and enzalutamide, PCT – paclitaxel, carboplatin, and panitumumab. RCB – Residual Cancer Burden[7]. TNBCtype4 – TNBC transcriptional subtype[16] classified into four categories: BL1 – basal-like 1, BL2 – basal-like 2, M – mesenchymal, LAR – luminal androgen receptor.

Supplemental Figure 2

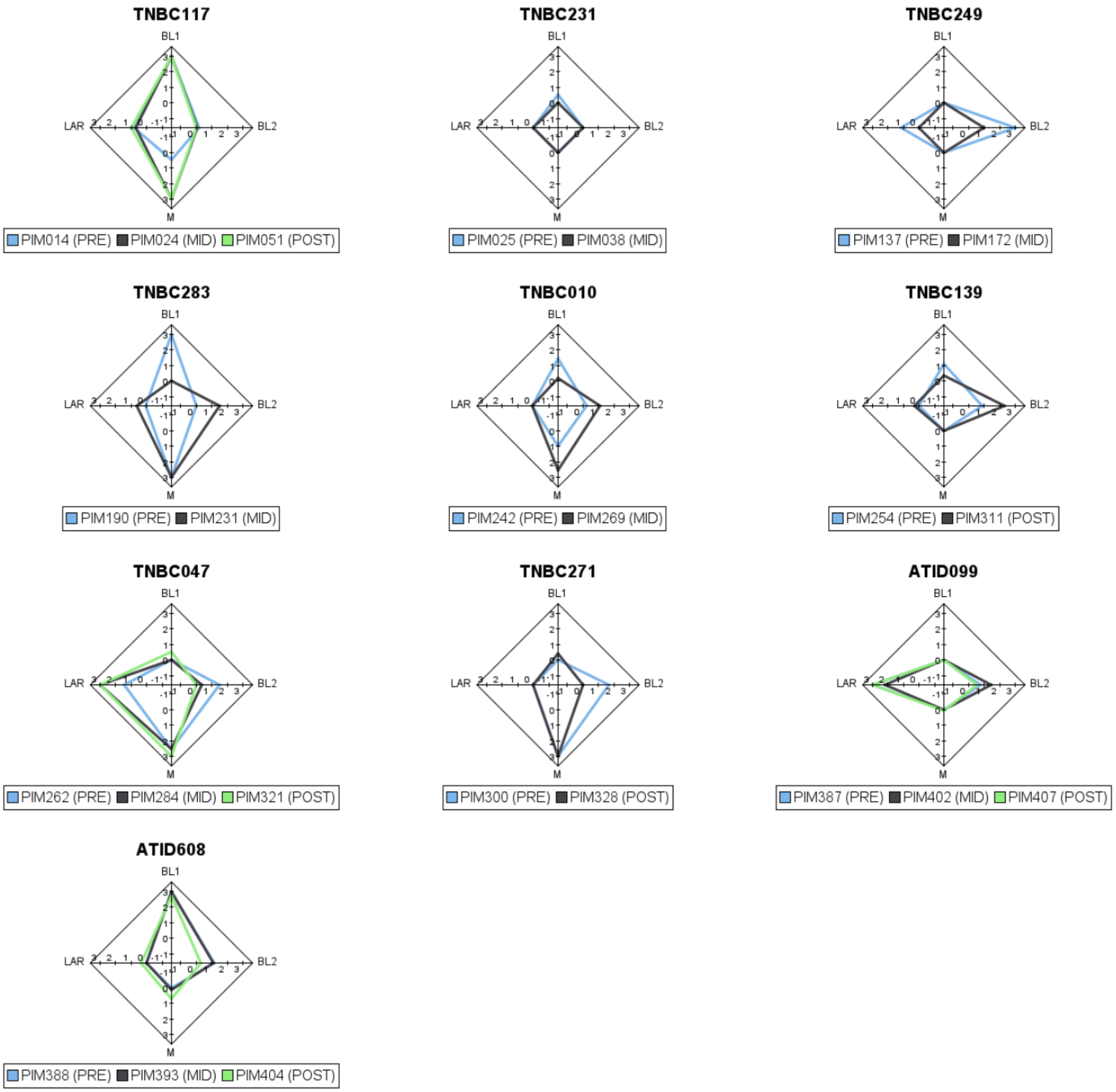

Supplemental Figure 2. Radar plots showing the -log$_{10}$(p-value) of TNBCtype4 classification (BL1, BL2, M, and LAR) for longitudinal sets. Patient identifier (PiD) is listed above.

Supplemental Figure 3

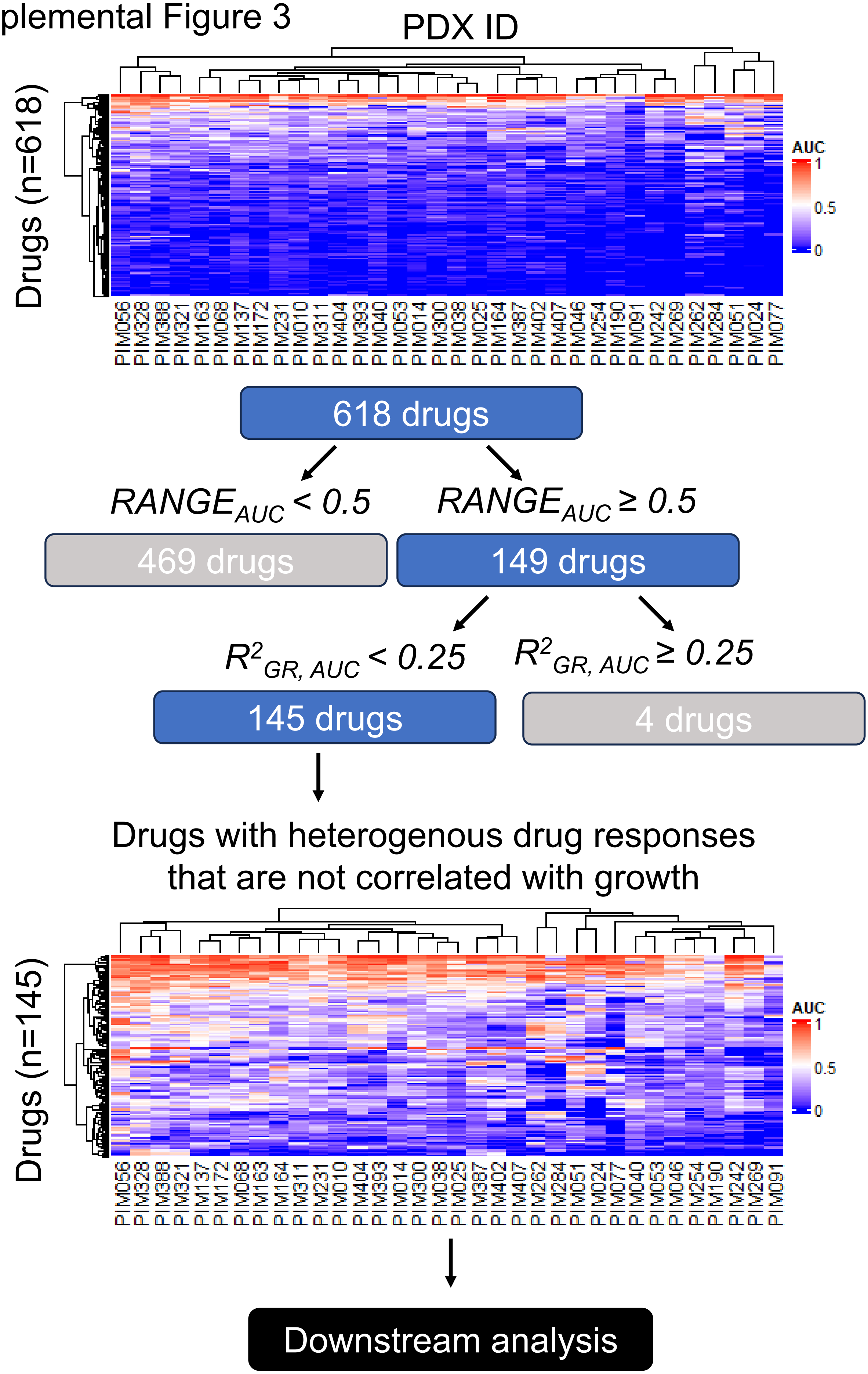

Supplemental Figure 3. Schematic of inclusion/exclusion criteria used for high throughput drug screening data. Top panel shows the heatmap of AUC values for all drugs versus PDX models (PDX ID). A flow chart shows the filtering steps from an initial library of 618 drugs to a final dataset of 145 drugs suitable for analysis. A range criterion and growth association ($R^2_{GR,\ AUC}$) were used to remove drugs likely to be confounded by drugs that did not exhibit a heterogeneous response (pan-active or pan-inactive) or differences in growth rate, respectively. Bottom panel shows the filtered heatmap of drugs that are used for downstream analysis.

Supplemental Figure 4

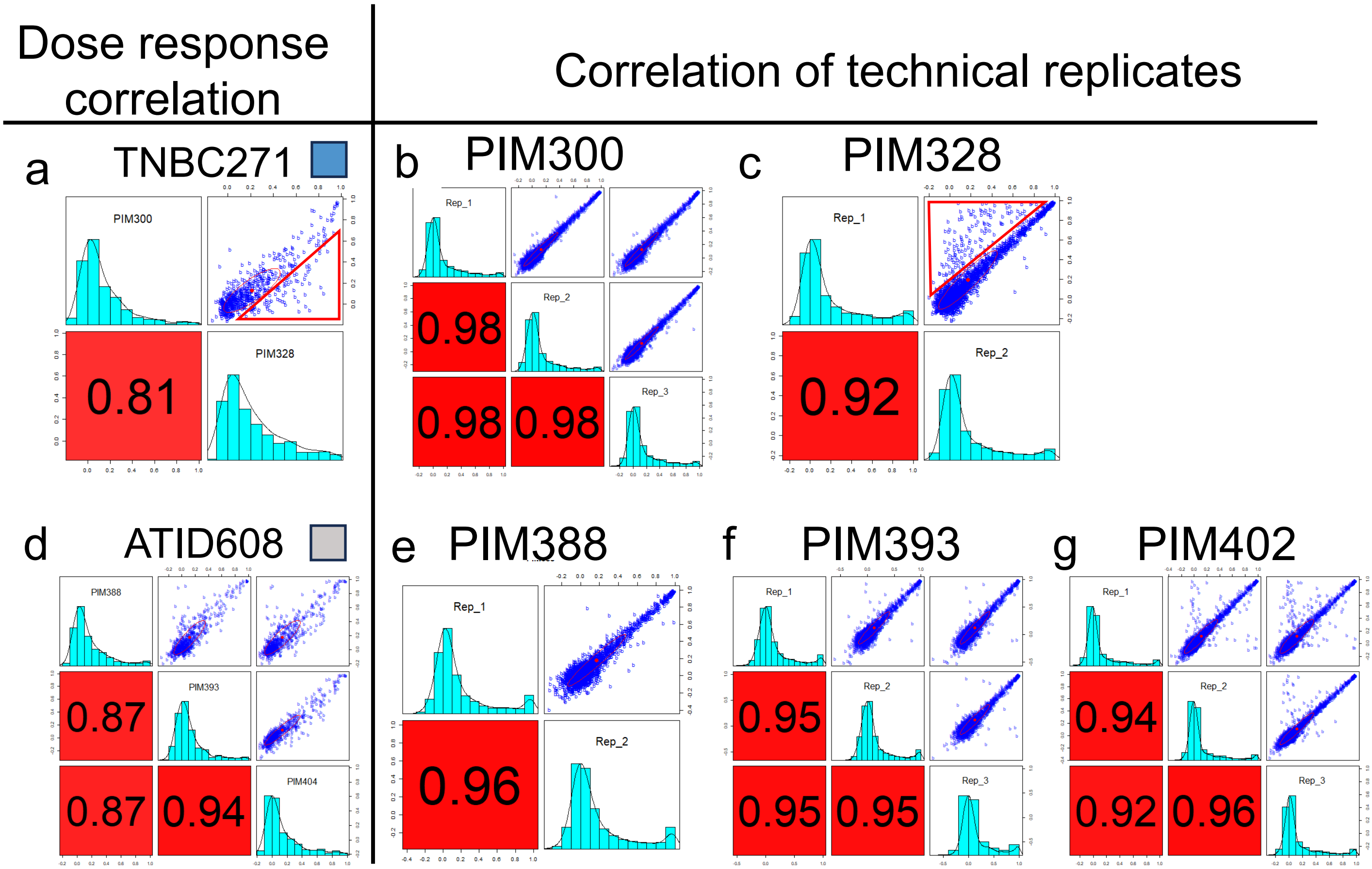

Supplemental Figure 4. Batch effect detection and replicate analysis for TNBC271 and AITD608. A, D) Pairs plot showing the distribution of AUC values for individual PDX models, the correlation between models, and the numerical Pearson correlation in red. B-C, E-G show pairs plots of the distribution of single replicates of FA-normalized CTG values, the correlation between off-plate technical replicates, and the numeric Pearson correlation (Red Block). Number of replicates (n = 2 or n=3) was determined by the availability of biological materials. Regions highlighted by red triangles show regions of dissimilarity that are likely driving divergent clustering.

Supplemental Figure 5

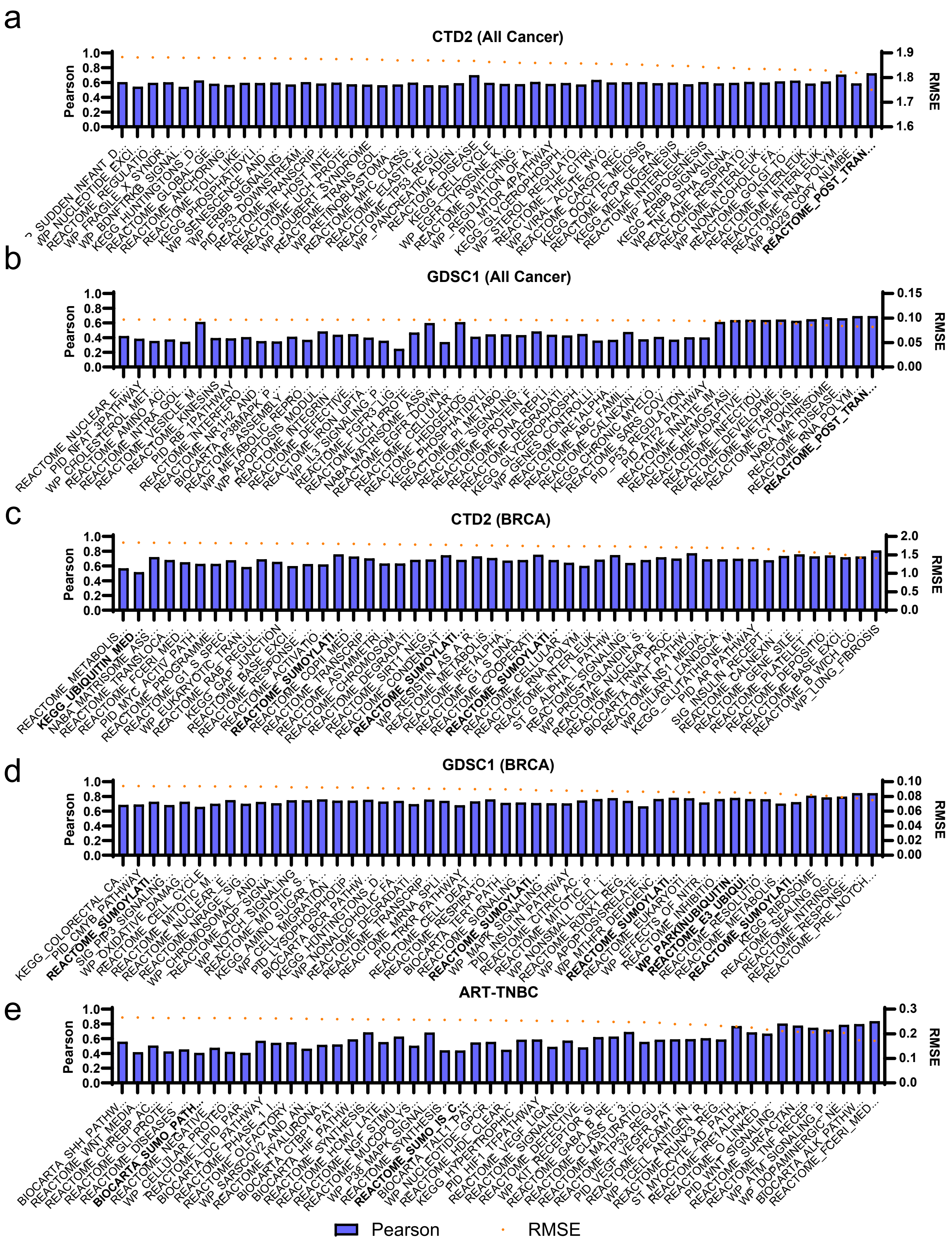

Supplemental Figure 5. Training metrics for L1-penalized (Lasso) regression models trained on each C2 canonical pathways' genes as input features. X-axis shows the name of the gene set. Bold font emphasizes ubiquitin-like post-translational modification pathways (i.e., ubiquitination, sumoylation, neddylation). Data shows the Pearson correlation (Left Y-axis, Blue bars) or root mean squared error (RMSE, Right Y-axis, Orange Dot) from the Leave-one-out-cross-validated (LOOCV) actual versus predicted values. Analysis was performed data from the A) Cancer Target Discovery and Development (CDT2) across all cancers, B) Genomics of Drug Sensitivity in Cancer (GDSC1), across all cancers, C) on CTD2 for only breast cancers (BRCA), D) on GDSC1 for only BRCA, and Ee) our ARTEMIS-TNBC PDX cohort.

Supplemental Figure 6

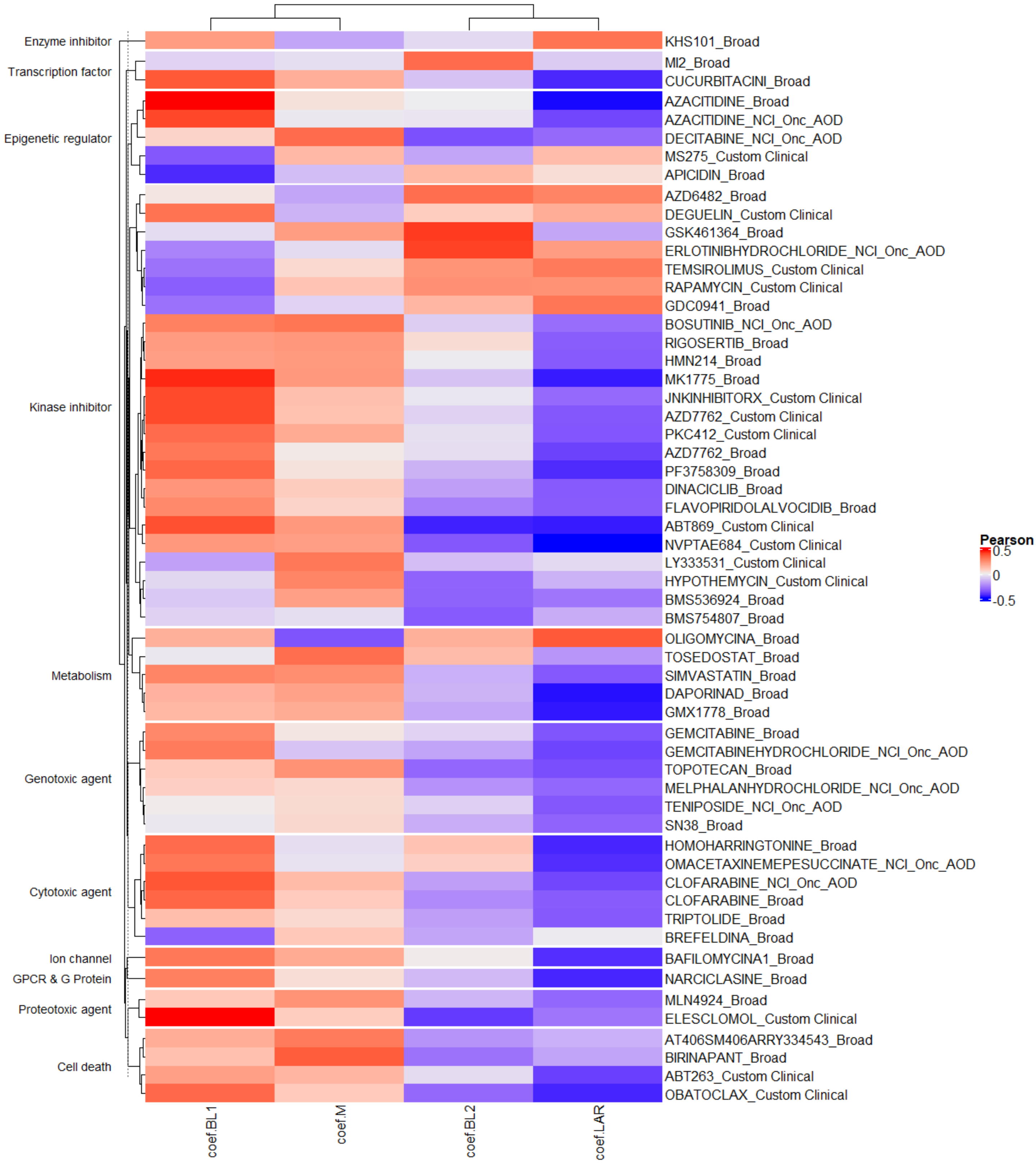

Supplemental Figure 6. Heatmap showing the correlation of drugs AUC to continuous TNBCtype4 fit coefficients. The data is filtered to only show the Pearson correlation of Drug AUC to TNBCtype4 using the following criteria: rowMax(|*Pearson*|) ≥ *0.3*.

Supplemental Figure 7

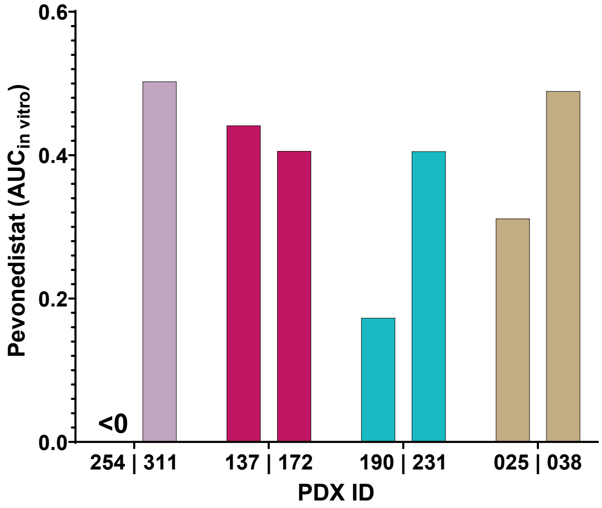

Supplemental Figure 7. Bar chart showing the AUC values from the *in vitro* HTS screens for PDX models selected for validation *in vivo*. Paired models are shown in the same color.

Supplemental Figure 8

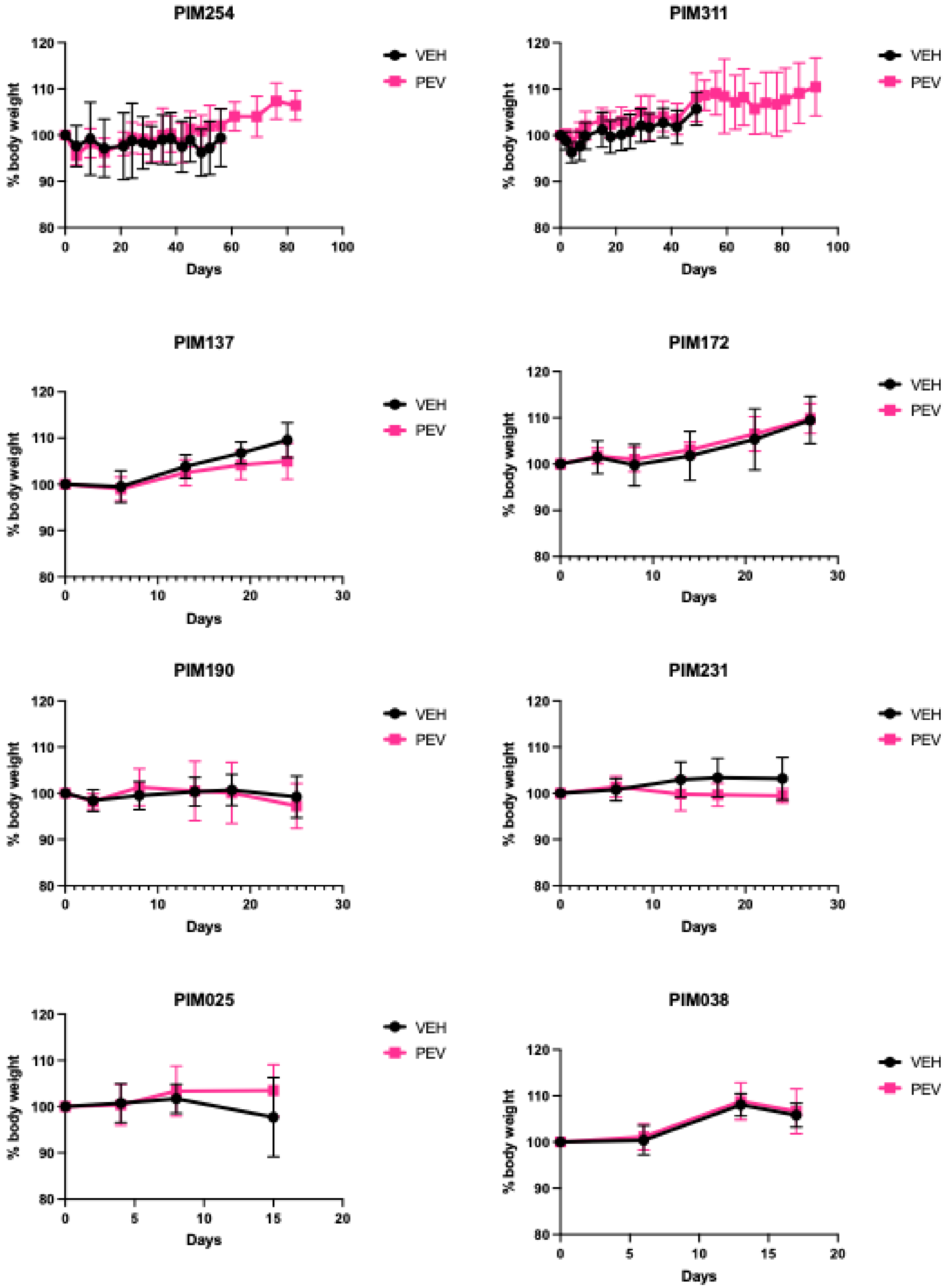

Supplemental Figure 8. Percent change in body weight over time (days) for mice treated with 60 mg/kg pevonedistat (PEV, pink) or vehicle treated control (VEH, black).

Supplemental Figure 9

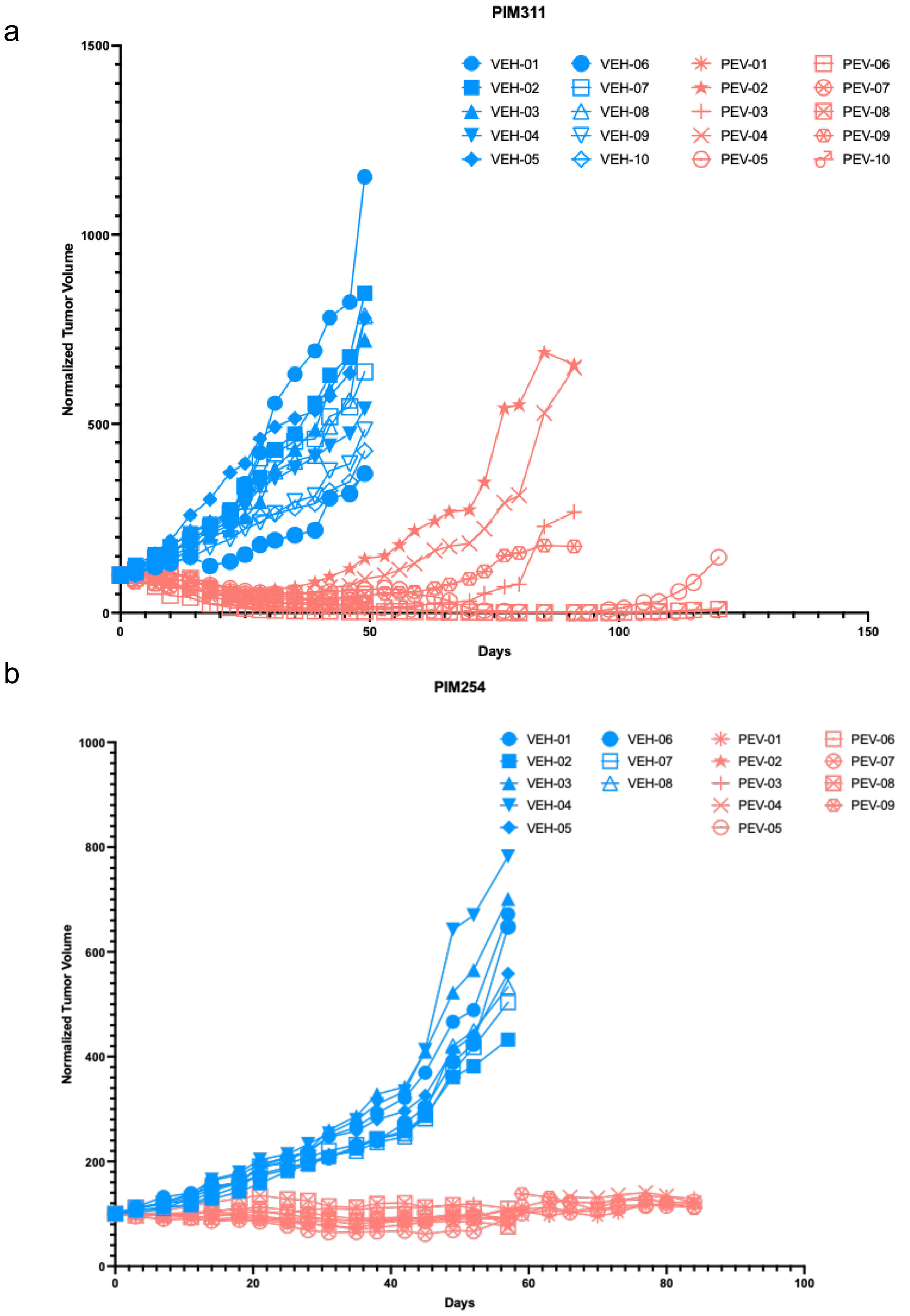

Supplemental Figure 9. Long term follow-up study of pevonedistat-responsive PDX models. Tumor volume was determined using caliper measurements and normalized to the starting volume (100%). Each line denotes an individual animal on either the vehicle (blue) or pevonedistat (red) arm. Data shows spontaneous resistance to pevonedistat in a subset of PIM311 mice after approximately two months of dosing.

Supplemental Figure 10

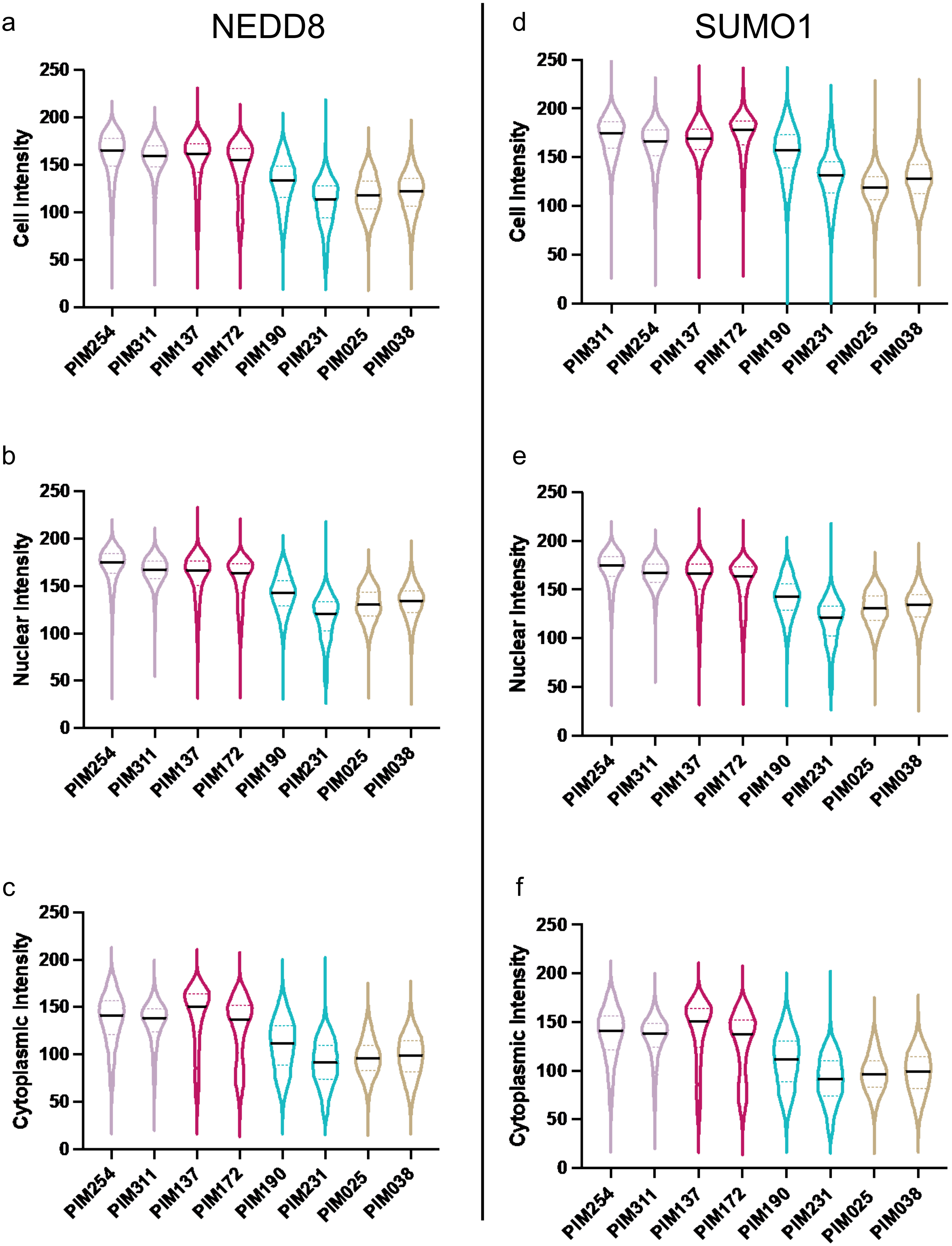

Supplemental Figure 10. Violin plot of the quantification of cellular (A), nuclear (B), or cytoplasmic (C) NEDD8 IHC intensity in PDX tumor cells and cellular (D), nuclear (E), or cytoplasmic (F) SUMO1 IHC intensity in PDX tumor cells. Paired models are shown in the same color. Images were quantified from three tumors per PDX model.

Supplemental Figure 11

Term_Description	Fold_Enrichment	occurrence	support	lowest_p	highest_p	Up_regulated	Down_regulated
Cell cycle	2.49	10.00	0.13	0.00	0.00	CCND2, E2F5, CDKN1B, CDK7, ANAPC4, ANAPC5, TTK, MAD1L1, EP300, PRKDC, ORC4	CDK6, E2F1, CDKN2C, RBX1, CDC25C, YWHAB, PKMYT1, CDC23, ANAPC15, SMC1B, GADD45B
Adherens junction	2.40	10.00	0.15	0.00	0.00	PARD3, CSNK2A1, CSNK2A3, SNAI1, EP300	ACTN4, CTNNA1, ACTB, RHOA, FYN, MAPK3
Axon guidance	1.86	9.00	0.03	0.00	0.02	NCK1, UNC5A, EPHA6, EPHB6, CXCR4, SLIT2, ROBO1, ROBO2, PLXNA1, ILK, CAMK2A, PIK3CA, PARD3	NTN1, TRPC1, FYN, RHOA, ROCK2, MYL9, MAPK3, SRGAP1, PRKCA, BMPR2
Pathogenic Escherichia coli infection	1.27	10.00	0.03	0.00	0.00	ABI1, MYO1B, MYO1G, TUBA3E, NCK1, TNFRSF10A, TNFRSF10B, NAIP, SEC24B	ACTB, RHOA, MYH9, ROCK2, FYN, CTTN, MAPK3, BAK1
Hippo signaling pathway	1.51	10.00	0.06	0.00	0.00	PARD3, LATS2, BMPR1A, WNT10B, WNT10A, CCND2	NF2, PPP2CA, DLG1, BIRC5, BMPR2, FZD7, YWHAB, APC, ACTB, CTNNA1
Natural killer cell mediated cytotoxicity	1.12	9.00	0.01	0.00	0.00	PTK2B, PIK3CA, TNFRSF10A, TNFRSF10B	MAPK3, FYN, SHC4, MICA, PRKCA, IFNAR2
Mitophagy - animal	1.82	10.00	0.02	0.00	0.00	MAP1LC3C, MAP1LC3B2, CSNK2A1, CSNK2A3, TBC1D15	UBB, BECN1, E2F1, MRAS
**Ubiquitin mediated proteolysis**	1.24	10.00	0.07	0.00	0.00	TRIM32, CUL2, ANAPC4, ANAPC5	UBB, UBE2A, UBE2D2, STUB1, RBX1, FBXO4, CDC23, ANAPC15
Nucleocytoplasmic transport	1.67	10.00	0.02	0.00	0.00	NUP98, TPR, TNPO1, IPO7, IPO11, SNUPN, XPO4, UPF2, THOC1	RAE1, SUMO3, EEF1A2
Fluid shear stress and atherosclerosis	1.77	10.00	0.02	0.00	0.02	GSTA5, THBD, PIK3CA, IL1R2, SELE, ACVR2B, BMPR1A	CALM2, CALML4, TRPV4, GSTP1, NQO1, PRKAA1, RHOA, ACTB, BMPR2, SUMO3

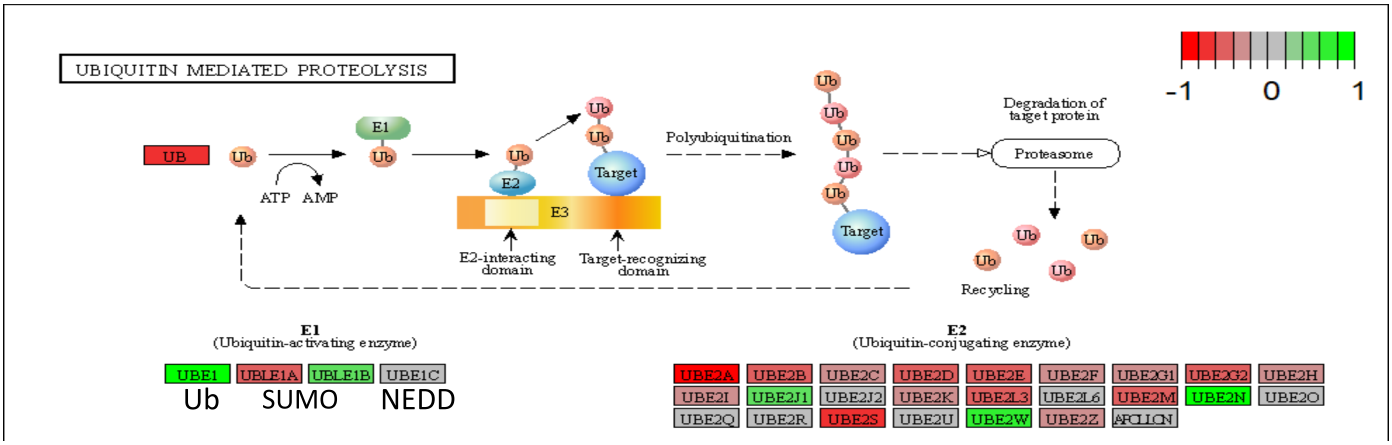

Supplemental Figure 11. Top) Top 10 enriched pathways from pathfinder analysis of *in vivo* drug response correlated genes. Term_Description – The name of the KEGG pathway. Fold_enrichment – The overall term enrichment term from pathfinder. Occurrence – the number of iterations in which the term was identified. Support – proportion of subnetwork leading to the enrichment within an iteration. Lowest_p and Highest_p – lowest and highest adjusted p-value term over all iterations of the enrichment analysis. **Bottom)** Color-coded KEGG overlay showing for the ubiquitin-mediated proteolysis pathway. Heat scale shows strength of the positive correlation (1, green) or negative correlation (-1, red).

Supplemental Figure 12

a

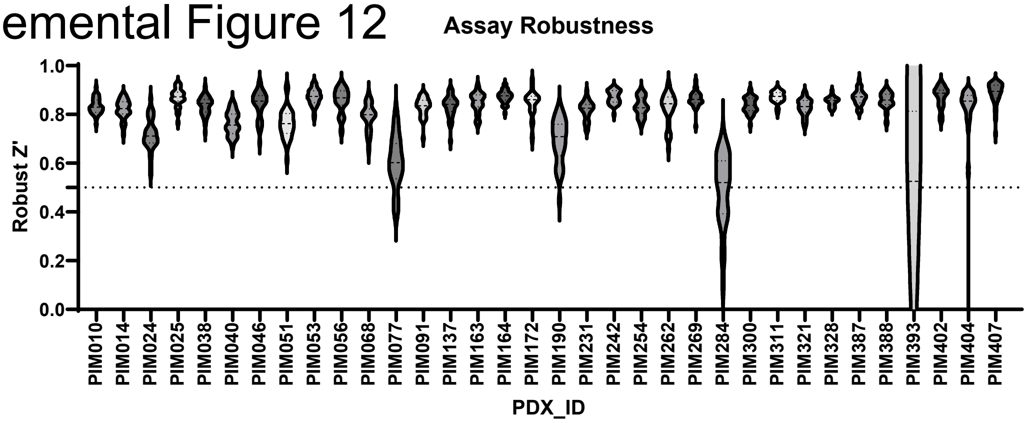

b

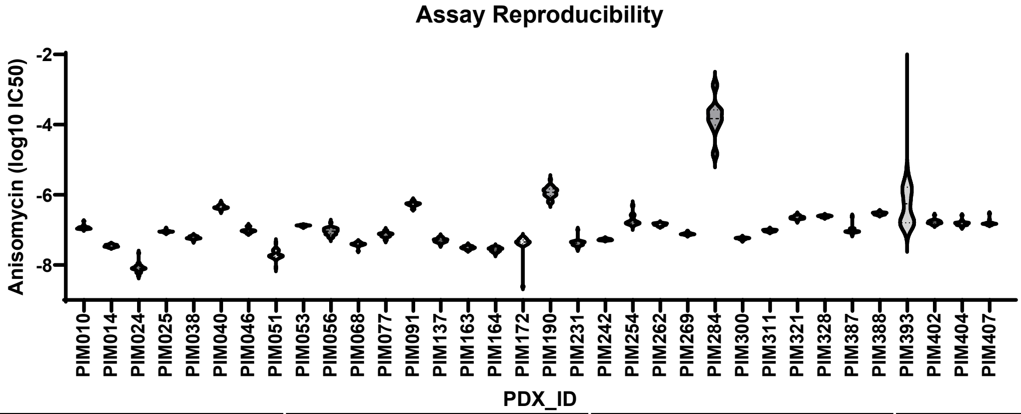

c

PDX ID	Robust Z' (Median)	Robust Z' (StdDev)	MSR
PIM010	0.83	0.03	1.33
PIM014	0.82	0.04	1.24
PIM024	0.71	0.06	2.04
PIM025	0.87	0.04	1.20
PIM038	0.85	0.04	1.29
PIM040	0.76	0.05	1.41
PIM046	0.85	0.06	1.46
PIM051	0.76	0.06	2.17
PIM053	0.87	0.03	1.15
PIM056	0.87	0.05	1.83
PIM068	0.80	0.05	1.38
PIM077	0.60	0.11	1.55
PIM091	0.84	0.04	1.47
PIM137	0.84	0.04	1.46
PIM163	0.86	0.04	1.30
PIM164	0.88	0.03	1.46
PIM172	0.86	0.05	>3
PIM190	0.71	0.09	2.57
PIM231	0.83	0.04	1.75
PIM242	0.87	0.03	1.15
PIM254	0.83	0.04	2.46
PIM262	0.84	0.06	1.24
PIM269	0.86	0.03	1.19
PIM284	0.52	0.18	>3
PIM300	0.84	0.03	1.20
PIM311	0.87	0.02	1.19
PIM321	0.83	0.03	1.34
PIM328	0.86	0.02	1.17
PIM387	0.87	0.03	1.97
PIM388	0.86	0.03	1.23
PIM393	0.53	2.11	>3
PIM402	0.89	0.04	1.44
PIM404	0.85	0.46	1.47
PIM407	0.89	0.05	1.61

Supplemental Figure 12. A) violin plot showing the distribution of robust z-prime (Z') values for each PDX model. B) The distribution of $\log_{10}(IC_{50}\ [M])$ for Anisomycin dose response curves to show high technical reproducibility. C) Statistics table summarizing the results presented in A and B.

Supplemental Figure 13

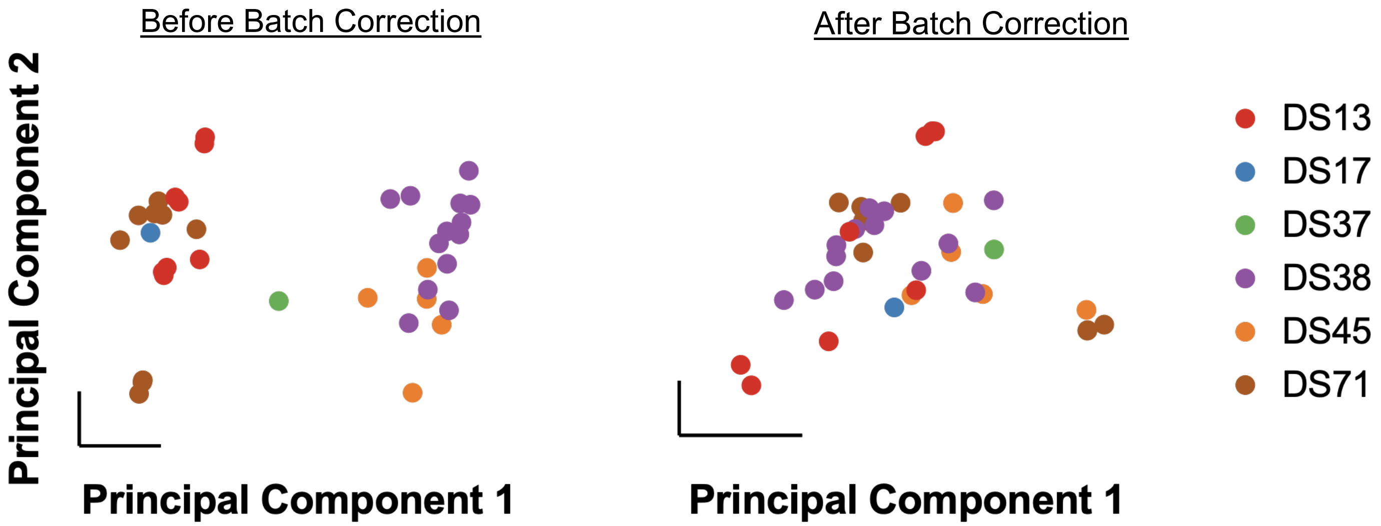

Supplemental Figure 13. These plots show the first two principal components (x- and y-axes) of the gene expression profiles of PDX tumors (dots). They were sequenced in separate batches, delineated by the colors. The gene expression profiles of the tumors before batch correction are shown in the left panel, and the ones after batch correction are shown in the right.

Supplemental Figure 14

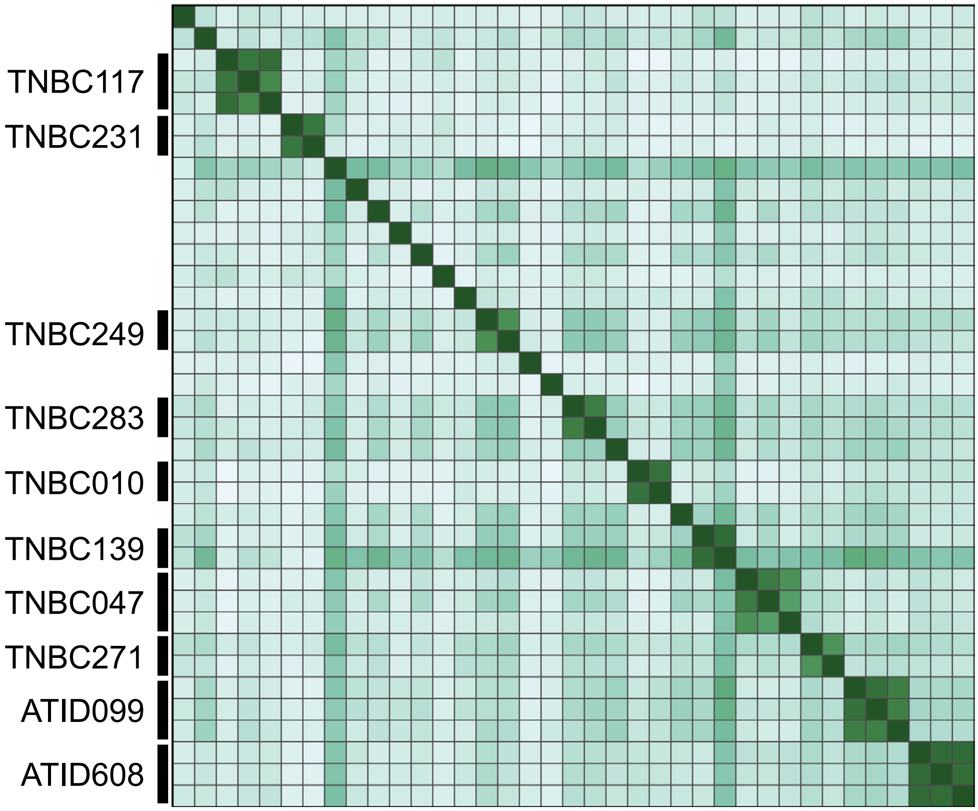

Supplemental Figure 14. This plot shows the similarity of the single nucleotide polymorphisms detected in the RNA-Seq profiles of the PDX tumors. The tumors are shown along the rows and the columns in the same order. Longitudinal tumors from the same patients are annotated. The colors show the correlations of the allele frequencies determined by NGSCheckMate. Deeper colors indicate higher correlations.
