# Supplementary material for: Targeting neddylation and sumoylation in chemoresistant triple negative breast cancer
Source: NPJ Breast Cancer. 2024 May 27;10:37. doi: 10.1038/s41523-024-00644-4 (PMC11130334; doi:10.1038/s41523-024-00644-4)
Supplement: Supplementary file 2 — Reporting Summary [file 41523_2024_644_MOESM2_ESM.pdf]

nature portfolio

Corresponding author(s): Helen Piwnica-Worms

Last updated by author(s): Mar 14, 2024

Reporting Summary

Nature Portfolio wishes to improve the reproducibility of the work that we publish. This form provides structure for consistency and transparency in reporting. For further information on Nature Portfolio policies, see our Editorial Policies and the Editorial Policy Checklist.

Statistics

For all statistical analyses, confirm that the following items are present in the figure legend, table legend, main text, or Methods section.

n/a	Confirmed	
☐	☒	The exact sample size (*n*) for each experimental group/condition, given as a discrete number and unit of measurement
☐	☒	A statement on whether measurements were taken from distinct samples or whether the same sample was measured repeatedly
☐	☒	The statistical test(s) used AND whether they are one- or two-sided<br>*Only common tests should be described solely by name; describe more complex techniques in the Methods section.*
☐	☒	A description of all covariates tested
☐	☒	A description of any assumptions or corrections, such as tests of normality and adjustment for multiple comparisons
☐	☒	A full description of the statistical parameters including central tendency (e.g. means) or other basic estimates (e.g. regression coefficient) AND variation (e.g. standard deviation) or associated estimates of uncertainty (e.g. confidence intervals)
☐	☒	For null hypothesis testing, the test statistic (e.g. *F*, *t*, *r*) with confidence intervals, effect sizes, degrees of freedom and *P* value noted<br>*Give P values as exact values whenever suitable.*
☒	☐	For Bayesian analysis, information on the choice of priors and Markov chain Monte Carlo settings
☒	☐	For hierarchical and complex designs, identification of the appropriate level for tests and full reporting of outcomes
☒	☐	Estimates of effect sizes (e.g. Cohen's *d*, Pearson's *r*), indicating how they were calculated

Our web collection on statistics for biologists contains articles on many of the points above.

Software and code

Policy information about availability of computer code

Data collection	HTS: Dassault Systemes (DS) BIOVIA Pipeline Pilot, BioTEK, . RNA-sequencing: Python, NGSCheckMate, FreeBayes, BETSY, R Statistics (limma, edgeR, Pathfinder); Image analysis: DS BIOVIA Pipeline Pilot, Python (CellPose), ilastik
Data analysis	Dassault Systemes (DS) BIOVIA Pipeline Pilot, Python/Anaconda, R/RStudio

For manuscripts utilizing custom algorithms or software that are central to the research but not yet described in published literature, software must be made available to editors and reviewers. We strongly encourage code deposition in a community repository (e.g. GitHub). See the Nature Portfolio guidelines for submitting code & software for further information.

Data

Policy information about availability of data

All manuscripts must include a data availability statement. This statement should provide the following information, where applicable:

- Accession codes, unique identifiers, or web links for publicly available datasets
- A description of any restrictions on data availability
- For clinical datasets or third party data, please ensure that the statement adheres to our policy

Curated data frames, open-source code, and interactive analysis (e.g., networks) are available without restriction at https://github.com/ReidTPowell/TNBC-PGx. Code was developed using R 4.3.1 using RStudio. Specific package requirements and version can be found in the R markdown notebook on the listed page. Analytical workflows generated in commercial/proprietary environments (BioVia Pipeline Pilot) are available upon request.

Research involving human participants, their data, or biological material

Policy information about studies with human participants or human data. See also policy information about sex, gender (identity/presentation), and sexual orientation and race, ethnicity and racism.

Reporting on sex and gender	This study focused on triple negative breast cancer, which mainly effects women. Accordingly, all PDX models were established from female breast cancer patients and maintained in female mice.
Reporting on race, ethnicity, or other socially relevant groupings	*Please specify the socially constructed or socially relevant categorization variable(s) used in your manuscript and explain why they were used. Please note that such variables should not be used as proxies for other socially constructed/relevant variables (for example, race or ethnicity should not be used as a proxy for socioeconomic status).*<br>*Provide clear definitions of the relevant terms used, how they were provided (by the participants/respondents, the researchers, or third parties), and the method(s) used to classify people into the different categories (e.g. self-report, census or administrative data, social media data, etc.)*<br>*Please provide details about how you controlled for confounding variables in your analyses.*
Population characteristics	*Describe the covariate-relevant population characteristics of the human research participants (e.g. age, genotypic information, past and current diagnosis and treatment categories). If you filled out the behavioural & social sciences study design questions and have nothing to add here, write "See above."*
Recruitment	*Describe how participants were recruited. Outline any potential self-selection bias or other biases that may be present and how these are likely to impact results.*
Ethics oversight	The ARTEMIS trial (NCT02276443) is overseen through an MD Anderson IRB-approved protocol (2014-0185).

Note that full information on the approval of the study protocol must also be provided in the manuscript.

Field-specific reporting

Please select the one below that is the best fit for your research. If you are not sure, read the appropriate sections before making your selection.

☒ Life sciences ☐ Behavioural & social sciences ☐ Ecological, evolutionary & environmental sciences

For a reference copy of the document with all sections, see nature.com/documents/nr-reporting-summary-flat.pdf

Life sciences study design

All studies must disclose on these points even when the disclosure is negative.

Sample size	Patient-derived models were developed on an opportunistic basis, no sample size criteria was imposed. For HTS, sample size was determined by optimizing the reproducibility of the signal. For in vivo studies, a sample size calculation was performed where we assumed a 2-sided 5% alpha and equal variances in the experimental groups. Under these assumption, we arrived at a sample size of n=8 mice per group could resolve a standard effect of 1.5 with 80% power.
Data exclusions	In some instances, based on when the average tumor size across the cohort was appropriate to begin dosing (100-150mm3), we had to exclude some tumors outside the size range or were able to exceed n=8 when more tumors fell within the range.
Replication	For HTS, two or three technical replicates were obtained across multiple assay plates, depending on the amount of material. Biological replicates of the complete screen were performed on a small number of PDX models to evaluate reproducibility across mouse passages.
Randomization	Once tumors reached ~100-150 mm3, they were randomized into treatment groups (vehicle or pevonedistat) such that both groups had a similar range of tumor sizes.
Blinding	Generally, tumor measurements were performed by an investigator blinded to the treatment group for each mouse, as dosing and analysis was performed by other investigators.

Reporting for specific materials, systems and methods

We require information from authors about some types of materials, experimental systems and methods used in many studies. Here, indicate whether each material, system or method listed is relevant to your study. If you are not sure if a list item applies to your research, read the appropriate section before selecting a response.

Materials & experimental systems

n/a	Involved in the study
☐	☒ Antibodies
☒	☐ Eukaryotic cell lines
☒	☐ Palaeontology and archaeology
☐	☒ Animals and other organisms
☒	☐ Clinical data
☒	☐ Dual use research of concern
☒	☐ Plants

Methods

n/a	Involved in the study
☒	☐ ChIP-seq
☒	☐ Flow cytometry
☒	☐ MRI-based neuroimaging

Antibodies

Antibodies used: NEDD8 (19E3) CST2754, 1:250 dilution; SUMO1 (no clone listed) CST4930, 1:50 dilution

Validation: Both antibodies have been validated for IHC by the manufacturer per their website. Recommended protocols were followed for antigen retrieval and antibody dilution. IHC results were compared to a no primary antibody control to determine background staining.

Animals and other research organisms

Policy information about studies involving animals; ARRIVE guidelines recommended for reporting animal research, and Sex and Gender in Research

Laboratory animals: PDX models were propagated in NOD/SCID (NOD.CB17-Prkdcscid/NcrCrl) mice (Charles River Laboratories, NCI colony). Tumors were implanted when mice were 4-8 weeks of age.

Wild animals: N/A

Reporting on sex: As this was a breast cancer study and all patient-derived materials were from female patients, all animal studies utilized female mice.

Field-collected samples: N/A

Ethics oversight: All experimental procedures were approved by the Institutional Animal Care and Use Committee (IACUC) at MD Anderson Cancer Center under IACUC protocol 00000978.

Note that full information on the approval of the study protocol must also be provided in the manuscript.

Plants

Seed stocks: *Report on the source of all seed stocks or other plant material used. If applicable, state the seed stock centre and catalogue number. If plant specimens were collected from the field, describe the collection location, date and sampling procedures.*

Novel plant genotypes: *Describe the methods by which all novel plant genotypes were produced. This includes those generated by transgenic approaches, gene editing, chemical/radiation-based mutagenesis and hybridization. For transgenic lines, describe the transformation method, the number of independent lines analyzed and the generation upon which experiments were performed. For gene-edited lines, describe the editor used, the endogenous sequence targeted for editing, the targeting guide RNA sequence (if applicable) and how the editor was applied.*

Authentication: *Describe any authentication procedures for each seed stock used or novel genotype generated. Describe any experiments used to assess the effect of a mutation and, where applicable, how potential secondary effects (e.g. second site T-DNA insertions, mosiacism, off-target gene editing) were examined.*
